# Induction of high titred, non-neutralising antibodies by self-adjuvanting peptide epitopes derived from the respiratory syncytial virus fusion protein

**DOI:** 10.1038/s41598-017-10415-w

**Published:** 2017-09-11

**Authors:** Noushin Jaberolansar, Keith J. Chappell, Daniel Watterson, Imogen M. Bermingham, Istvan Toth, Paul R. Young, Mariusz Skwarczynski

**Affiliations:** 1 School of Chemistry and Molecular Biosciences, The University of Queensland, St Lucia, Queensland, Australia; 2 Australian Infectious Diseases Research Centre, The University of Queensland, St Lucia, Queensland, Australia; 3 School of Pharmacy, The University of Queensland, Woolloongabba, Queensland, Australia

## Abstract

Respiratory syncytial virus (RSV) causes severe lower respiratory tract illness in infants and young children. The significant morbidity and mortality rates associated with RSV infection make an effective RSV vaccine development a priority. Two neutralising antibody binding sites, Ø and II, located on the pre-fusion RSV F glycoprotein are prime candidates for epitope-focused vaccine design. We report on a vaccine strategy that utilises a lipid core peptide (LCP) delivery system with self-adjuvanting properties in conjunction with either the antigenic site Ø or II (B cell epitopes) along with PADRE as a T helper cell epitope. These LCP constructs adopted the desired helical conformation in solution and were recognised by their cognate antibodies D25 and Motavizumab, specific for site Ø and II on RSV F protein, respectively. The LCP constructs were capable of eliciting higher levels of antigen specific antibodies than those induced by antigens administered with complete Freund’s adjuvant, demonstrating the potent adjuvanting properties of LCP delivery. However, the antibodies induced failed to recognise native F protein or neutralise virus infectivity. These results provide a note of caution in assuming that peptide vaccines, successfully designed to structurally mimic minimal linear B cell epitopes, will necessarily elicit the desired immune response.

## Introduction

Human respiratory syncytial virus (RSV) causes serious lung and airway infection such as pneumonia and bronchiolitis in infants and young children^1^. Almost all children are infected with RSV by age two and infections recur throughout life^2^. RSV infections can also be serious in the elderly, adults with certain heart and lung problems and immunocompromised populations^3^. The significant mortality and morbidity rates of RSV infection emphasise the urgency of RSV vaccine development.

The first attempt to produce a vaccine against RSV, using formalin-inactivated, alum precipitated RSV (FI-RSV), failed to protect children upon RSV infection and in fact caused more pronounced disease in some instances^4^. Subsequently, various approaches have been developed with the aim of eliciting a protective immune response without priming for enhanced disease^5^. These new designs have been facilitated by recent developments in both vaccine technology and a more detailed understanding of virus biology and a protective immune response at the molecular level. Much of this research has focused on the RSV fusion (F) protein, as it is the most highly conserved viral surface protein^6, 7^ and is the primary determinant of neutralising antibodies^8–11^. Multiple antigenic sites within F have been identified, determined through a variety of methods including peptide screening, analysis of resistant viruses and structural determination of antibody fragment (Fab) and F protein complexes^12^. Of these, two main sites have been the focus of epitope-based vaccine design and are designated as antigenic site Ø and site II. Site II was the first to be structurally defined and is the binding site for the therapeutic monoclonal antibody (mAb) Palivizumab and its affinity-optimised derivative, Motavizumab^13, 14^. The second, more recently identified, antigenic site Ø is the binding site for highly neutralising mAb D25^10^. This site is present at the apex of the pre-fusion conformation and is conformationally obscured in the post-fusion conformation.

While some subunit-based and live attenuated vaccines have shown promise in clinical trials^15^, an alternative, minimalistic approach to subunit-based vaccines is epitope-focused vaccine design. Such an approach focuses on only a single protective, neutralising epitope with the aim of eliciting known protective antibodies without inducing imbalanced T-cell responses.

Various attempts have been made to exploit antigenic site II in order to generate an RSV vaccine. Initial approaches produced humoral responses in mouse models that poorly recognised native RSV and demonstrated no neutralising activity^16, 17^. Subsequently, Correia and co-workers reproduced this site on a genetically encoded protein scaffold in order to enhance conformational stability^18^. Interestingly, while the scaffolded immunogens failed to produce a neutralising response in rodents, the majority of vaccinated macaques produced RSV neutralising antibodies. More recently, other groups have demonstrated that the site II epitope presented on virus-like particles or antibody based scaffolds can induce a neutralising antibody response in murine models^19, 20^. In comparison, due in part to its more recent discovery, site Ø has not yet been directly targeted by peptide-based vaccine design. However, this epitope has been a major focus in the development of stabilised pre-fusion F vaccine constructs^21^ and the virus neutralising effect of antibodies targeting this site has been shown to be significantly more potent than antibodies with comparable affinities to elsewhere within RSV F^10, 22–24^.

**Figure 1:**
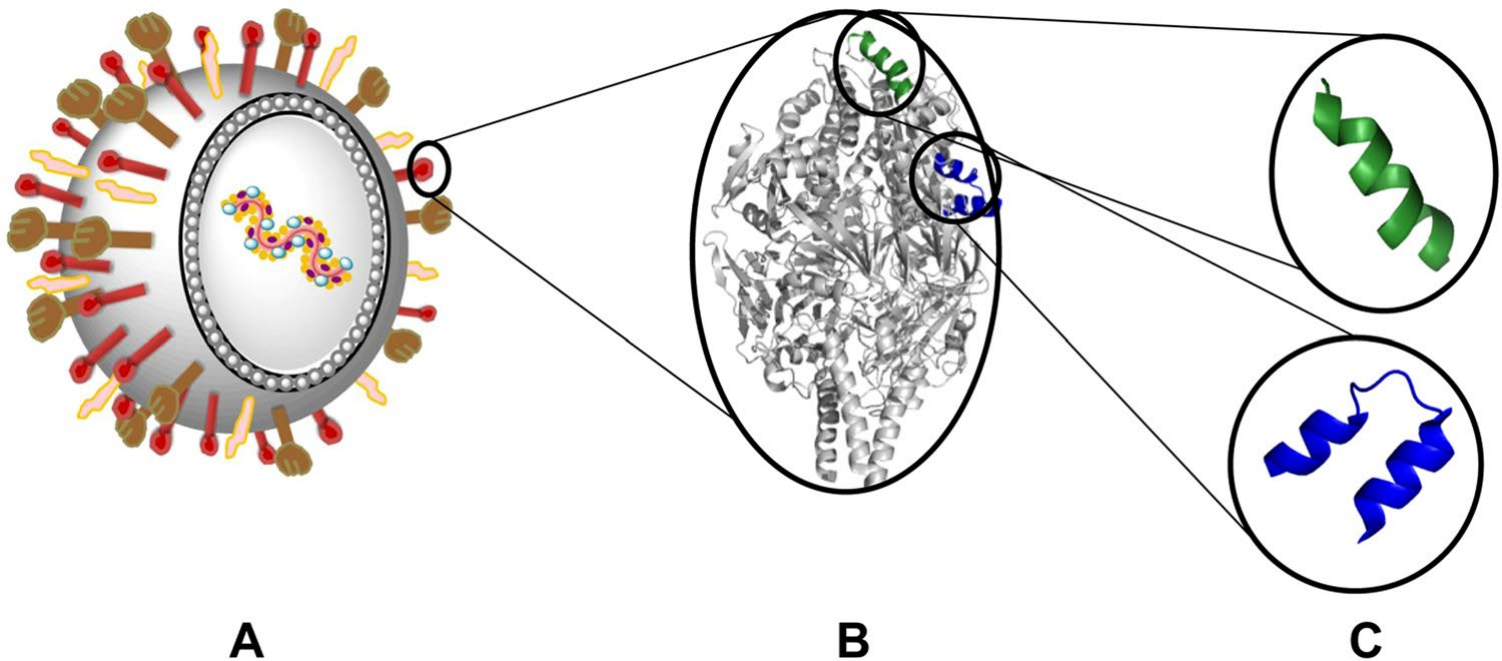
Schematic presentation of RSV vaccine design from whole pathogen to peptide-based vaccine. **(A)** whole pathogen, **(B)** F protein-based vaccine and **(C)** peptide-based vaccine candidates based on site Ø (green; aa196–211) and site II (blue; aa255–275).

**Figure 2:**
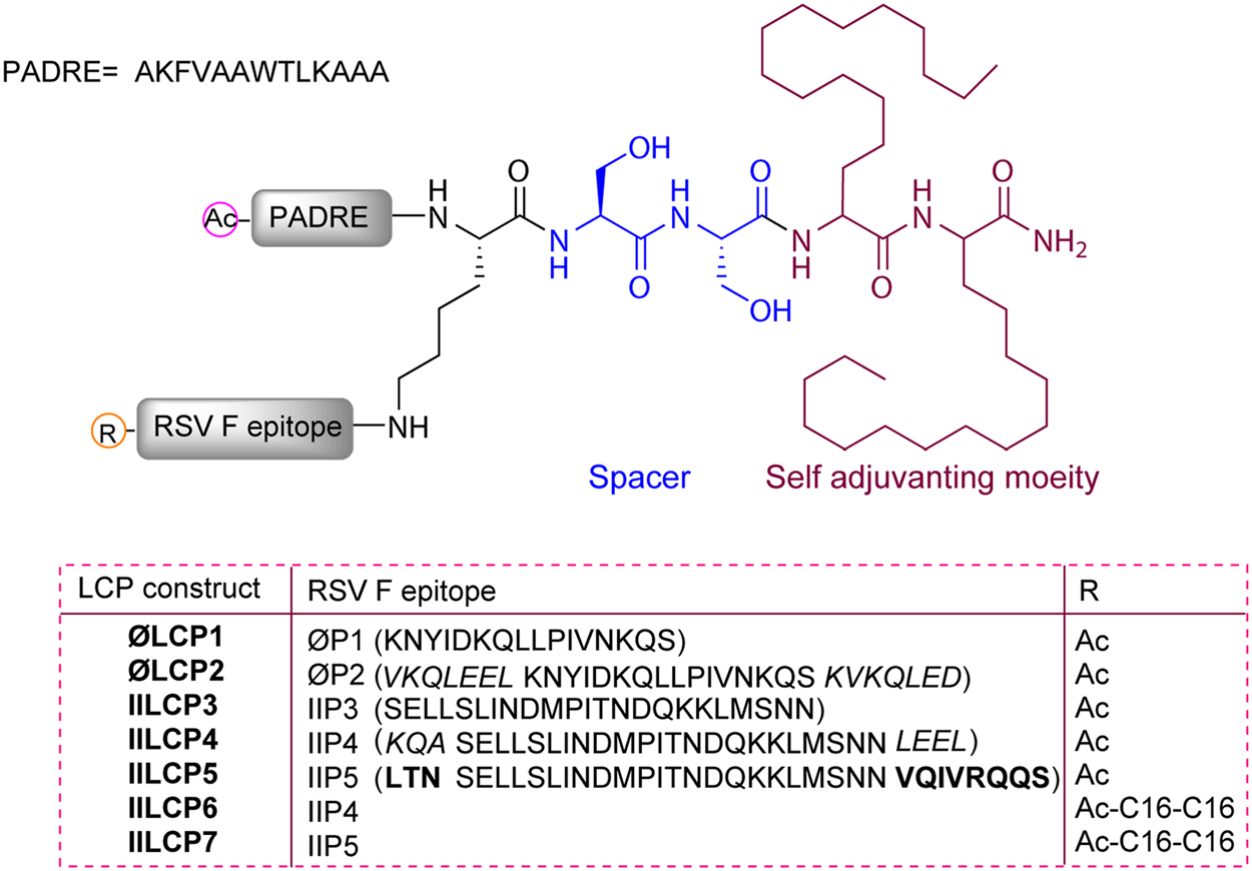
Overview of lipid core peptide-based vaccines candidate design. Structures of lipid core peptide vaccine candidates comprising self-adjuvanting Lipoamino acids, PADRE and RSV peptides. **ØP1** is the native sequence corresponding to epitope Ø (aa196–211) and **IIP3** is the native sequence corresponding to epitope II (aa255–275). **ØP2** and **IIP4** also correspond to the epitope Ø and II, respectively but are flanked by coiled-promoting sequence from GCN4 protein (italic fonts). **IIP5** is an extended sequence corresponding to epitope II (aa252–283); additional sequence (bold font). Control vaccine formulation includes non-covalently linked peptides comprising peptides (**P1**-**5**) physically mixed with PADRE and Complete Freund's Adjuvant (CFA).

In this study, we generated peptide-based RSV vaccine constructs with self-adjuvanting ability containing B-cell epitopes from either antigenic site II or site Ø. In general, peptide-based subunit vaccines contain the minimal component of a pathogen necessary to trigger effective immune responses (Fig. 1). Compared with whole pathogen strategy (attenuated or inactivated vaccines), synthetic peptides have several advantages with regard to cost and production feasibility^25, 26^. However, poor immunogenicity is one of the drawbacks of peptide-based vaccines and necessitates the help of an adjuvant to elicit a robust immune response. Lipid and lipopeptide conjugation is one approach that has been shown to improve immunogenicity of peptides^27–31^. We have previously developed the lipid core peptide (LCP) system, which is a self-adjuvanting delivery system for peptide vaccines that consists of a lipid core, a carrier or branching moiety and the peptide epitopes (Fig. 2)^30, 32^. The generated lipopeptides were able to activate immune cells through toll-like receptor-2 (TLR2), thereby enhancing the immunogenicity of incorporated antigens^33^. LCP delivery system has previously been utilised as a part of candidate vaccine formulation against several diseases^34–37^. In this study we utilised the LCP system to present RSV F-protein derived B-cell epitopes as novel RSV vaccine candidates. Peptides (**ØP1,2** and **IIP3-5**) were designed based on the antigenic site Ø and II using bioinformatic analysis. Subsequently, designed peptides were incorporated to the LCP delivery system by solid phase peptide synthesis (SPPS) and their immunological properties were assessed.

## Results

### Synthesis and characterisation of peptide epitopes and vaccine candidates

B-cell epitopes from two antigenic sites from the RSV F protein (II and Ø) were chosen for peptide-based RSV vaccine development. Both chosen sequences adopt an α-helical secondary structure within the native RSV F structure (Fig. 1). Short peptides were generated based on these sites (**ØP1** and **IIP3**). However, as short peptides, it is unlikely that these sequences will adopt their native configuration^37^. Thus, to promote helicity we also generated constructs (**ØP2** and **IIP4**) that were flanked by the yeast GCN4 coil promoting sequence^38^ at both N-terminus and C-terminus. An extended version of site II was also generated (**IIP5**). *In silico* analysis of secondary structure propensity of the designed constructs was performed using three different online structure prediction platforms (Porter, PSIPRED and Scratch) and then compared to available crystal structure^10^ (PDB ID: 4JHW). Modified peptides (**ØP2, IIP4** and **IIP5**) were predicted to adopt similar conformations as selected epitopes within the RSV F protein. All synthesised peptides were also incorporated into LCP system as previously described^30, 32^. The generated constructs (**ØLCP1,2** and **IILCP3-5**) consisted of the individual peptides along with the self-adjuvanting lipoamino acid and the T helper epitope PADRE (see Fig. 2). Two further constructs were generated (**IILCP6** and **IILCP7**), which consisted of peptides (**IIP4** and **IIP5**) fused to the LCP system but with two extra copies of lipoamino acid coupled at the N-terminus of synthesised B cell epitope to adopt helix-loop-helix conformation.

All peptides and LCP constructs were successfully synthesised by microwave-assisted SPPS using either Fmoc- or Boc-chemistry according to our standard procedures^32, 45^. All constructs were purified by HPLC and the structure was confirmed by ESI-MS. Dynamic light scattering (DLS) was performed to measure the particle size and polydispersity. **ØLCP2** and **IILCP4** formed smaller particles in 1% DMSO in PBS mixture (Table 1) compared to other constructs.

**Table 1:** Biophysical characterisation of vaccine candidates.

Candidate	Epitope	Additional sequences	R^*^	LAA^**^	Secondary structure (Determined by CD)	D25 k_D_ (nM)	Mota k_D_ (nM)	Diameter DLS (nm)
**ØP1**	Site Ø	—	Ac	—	Random coil	NB	NB	N/A
**ØP2**	Site Ø	GCN4	Ac	—	Random coil	~720	NB	N/A
**IIP3**	Site II	—	Ac	—	Random coil	NB	NB	N/A
**IIP4**	Site II	GCN4	Ac	—	Random coil	NB	NB	N/A
**IIP5**	Site II	Extended	Ac	—	Random coil	NB	~22	N/A
**ØLCP1**	Site Ø	—	Ac	2	Mixture of β-sheet, α-helix	NB	NB	225±10
**ØLCP2**	Site Ø	GCN4	Ac	2	α-helix	66	NB	40±3
**IILCP3**	Site II	—	Ac	2	β-sheet	NB	NB	2154±300
**IILCP4**	Site II	GCN4	Ac	2	α-helix	NB	~4	122±20
**IILCP5**	Site II	Extended	Ac	2	α-helix	NB	~15	181±25
**IILCP6**	Site II	GCN4	Ac-C16-C16	4	N/A	NB	~40	419±70
**IILCP7**	Site II	Extended	Ac-C16-C16	4	N/A	NB	~14	261±20

*Ac: acetylated amino acid (see Fig. 2).

**LAA: Lipoamino acid.

Circular dichroism (CD) analysis was performed to assess the adopted secondary structure of peptides and vaccine candidates (Supplementary Fig. S16). While all peptides (**ØP1,2 and IIP3**-**5)** showed random coil conformation with negative bands at λ = 200 nm, **ØLCP2, IILCP4** and **IILCP5** showed the desired α-helix properties, with a strong minimum at λ= 208 nm and a shallow minimum at λ = 219–222 nm. **ØLCP1** showed a mixture of β-sheet and α-helix with a minimum at λ= 209 nm, whereas **IILCP3** adopted β-sheet structure with a minimum at λ= 216. Spectra for **IILCP6** and **IILCP7** were not detected due to aggregation of the compounds under measurement condition.

**Figure 3:**
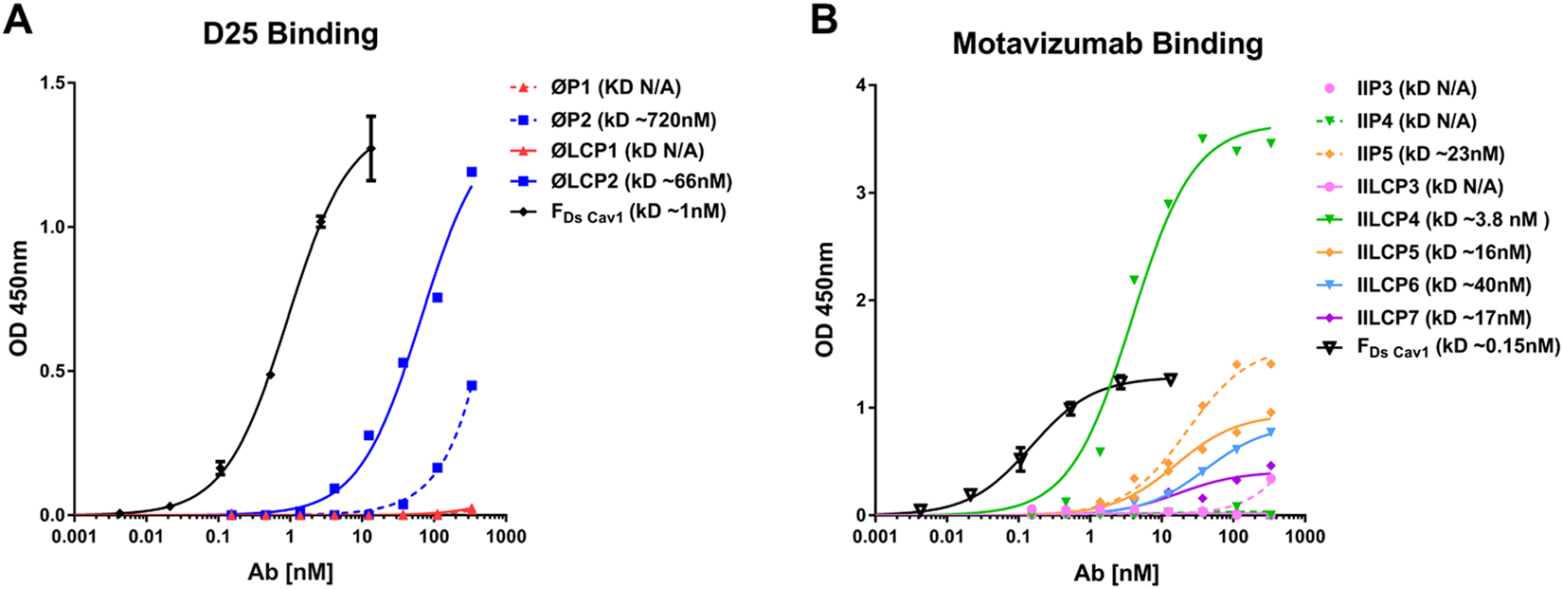
Binding avidity of monoclonal antibodies against vaccine candidates. Antibody binding assessed by serial dilution of antibody on ELISA plates were coated with vaccine constructs and curve fitting by non-linear regression with a one site specific binding model using Graphpad Prism 7 software. (**A**) D25 antibody recognised **ØLCP2** (k_D_: 66 ± 014 nM) and to a lesser extent **ØP2** (k_D_: 720 ± 036nM). Binding to F_Ds Cav1_ used as positive control (k_D_: 0.92 ± 00.08 nM). Motavizumab recognised **IILCP4** (k_D_: 3.8 ± 00.9nM), **IILCP5** (k_D_: 16 ± 03nM), **IILCP7** (k_D_: 17 ± 011nM), **IIP5** (k_D_: 23 ± 05nM) and to a lesser extent **IILCP6** (k_D_: 40 ± 04nM). Binding F_Ds Cav1_ was used as a positive control (k_D_: 0.15 ± 00.01nM).

We further tested the ability of site II and site Ø specific antibodies (Motavizumab and D25, respectively) to recognise the peptides and LCP constructs. Dissociation constant (k_D_) values were derived from a saturation one-site model. D25 clearly recognised the **ØLCP2** (66 ± 14 nM) but not **ØLCP1** (Fig. 3A). In comparison, in our analysis D25 bound to the native pre-fusion RSV F (F_Ds-Cav1_) with approximately 60-fold stronger avidity (0.92 ± 0.08 nM). Motavizumab bound **IILCP4** with the highest avidity (3.8 ± 0.9 nM), while in our analysis Motavizumab bound F_Ds-Cav1_ with approximately 25 fold stronger avidity (0.15 ± 0.01 nM). Motavizumab also recognised **IILCP3**, **5**, **6** and **7** and **IIP5** (Fig. 3B). In general binding avidity was stronger for LCP constructs compared to original peptides.

### Immunisation and Antigenicity

Due to the hydrophobic nature of the LCP vaccine candidates, **ØLCP1,2 and IILCP3-7** were dissolved in 1% DMSO for vaccine delivery. We did not anticipate any adverse effects due to the presence of DMSO as previous vaccine candidates have been administered *in vivo* with concentrations of DMSO as high as 10% without any adverse effect and it has also been shown that DMSO is not immunogenic^49, 50^. Mice were immunised by subcutaneous administration of vaccine candidates followed by three booster immunisations.

**Figure 4:**
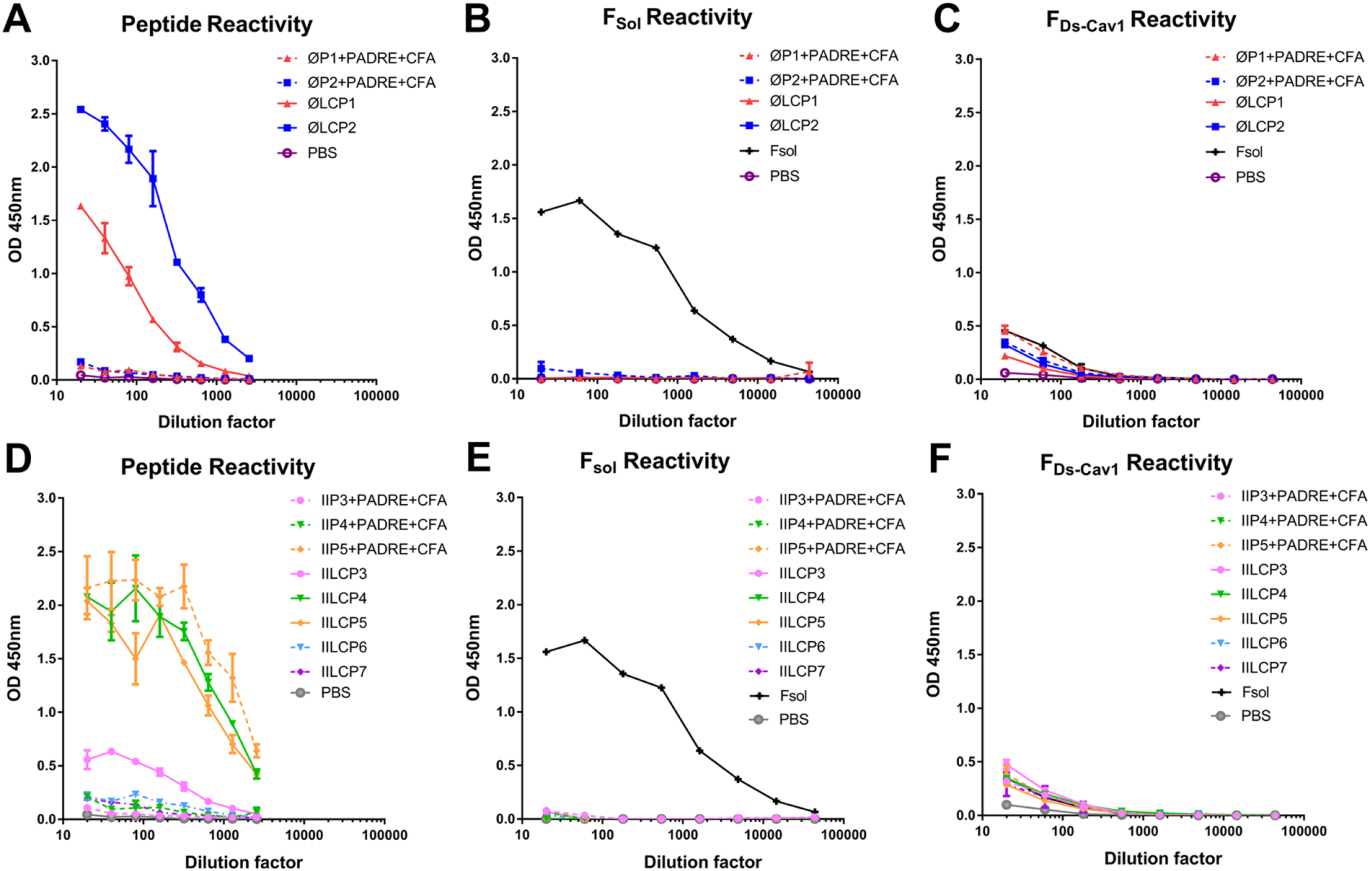
Specificity of immune response. Sera from vaccinated mice was used on ELISA plates coated with peptide and LCP constructs **(A** and **D)**, Fsol **(B** and **E)** and F_Ds-Cav1_ **(C** and **F)**. Bound IgG was detected by HRP-conjugated either anti-mouse or anti-human IgG antibodies on coated plate. Shown are the arithmetic mean averages of the ODs of duplicates with error bars indicating SD.

IgG antibodies titres in sera were determined for each group against the relevant antigen using ELISA. For vaccine candidates targeting site Ø, binding was only observed for the lipid core presented constructs, **ØLCP1** and **ØLCP2**, with **ØLCP2** showing the highest induction of a specific IgG response (Fig. 4A). Among vaccine candidates containing antigenic site II, sera from **IILCP4** and **IILCP5** and **IIP5** immunised animals showed higher specific IgG titres against matched antigen (Fig. 4D).

We then assessed the ability of our peptide-based vaccine candidates to elicit a response against native F protein using ELISA. In these experiments we used both F_sol_^51^ and F_Ds-Cav1_^21^, representing the post-fusion and stabilised pre-fusion form of F protein. The pre and post-fusion conformations had been previously validated by D25 and Motavizumab recognition (Fig. 3). All sera from vaccine candidates targeting antigenic site Ø and II showed no reactivity directed against F_sol_ (Fig. 4B,E) and limited reactivity against F_Ds-Cav1_, only slightly above the PBS control group (Fig. 4C,F). In contrast, sera from vaccinated mice with F_sol_ showed high level reactivity with F_sol_ protein (Fig. 4B,E)

Binding of vaccinated mice sera to RSV infected cells was also examined by immunofluorescence staining. Consistent with the ELISA data, only sera from F_sol_-vaccinated mice was able to detect RSV F in fixed infected cells, while sera from mice immunised with peptide-based vaccine candidates was unable to detect the RSV F antigen (Supplementary Fig. S17).

After investigating the immunogenicity of test sera via ELISA, the generation of a neutralising antibody response was assessed by plaque reduction neutralisation test (PRNT). Assays were performed with dilutions of pooled, heat-inactivated sera from each immunised group. No neutralisation was observed for any of the vaccine candidates or the control immunisation with F_sol_ (Supplementary Fig. S18). In comparison, potent neutralisation was observed using a purified chimeric recombinant anti-F antibody (Motavizumab).

## Discussion

RSV infection remains the leading cause of hospitalisation for infants under 5 years old. Despite decades of effort, an effective vaccine is not yet clinically available. This pressing need has impelled vaccine researchers to evaluate a wide variety of novel vaccine strategies for RSV infection. Among these approaches, subunit vaccines are an attractive option, with the potential to deliver epitope targeted responses without the safety concerns associated with live vaccines. However, peptide- and protein-based subunit vaccines are poorly immunogenic on their own and usually necessitate the addition of immunostimulatory agents to elicit the effective immune response. To overcome these challenges we have developed a novel self-adjuvanting LCP delivery system that is able to boost immune responses to incorporated peptide epitopes^44, 52, 53^. The use of a self-adjuvanting delivery system eliminates the requirement for vaccine formulation that incorporates an adjuvant such as complete Freund’s adjuvant (CFA) and can aid in the correct presentation of the incorporated epitope in the desired conformation^34, 36^. In this study, we investigated the use of the LCP delivery system (Fig. 2) in the development of novel peptide based subunit RSV vaccines.

The main goal of most of RSV vaccines is to block the virus entry either through preventing attachment or arresting membrane fusion. Blocking of virus entry is a reported mechanism of neutralising antibodies in protection against most of viruses^54^. Stabilising the conformation of desired neutralising epitopes within the F protein in the native pre-fusion conformation is paramount for effective RSV epitope-focused vaccine development. This is highlighted by the finding that pre-absorption of human sera with post-fusion F protein fails to remove neutralising activity^11^. Furthermore, the identification of highly neutralising pre-fusion specific antibodies such as D25 and the corresponding recognition site Ø provides a structural target for vaccine development. With this in mind, we chose to examine this site along with the well-established antigenic site II for the basis of our LCP-based vaccine candidates.

In the pre-fusion structure of RSV F, the Ø and II epitopes are mainly α-helical in nature, with the primary determinate of site Ø a single helix structure comprising residues 196–211 and site II present as a helix-loop-helix motif made up of residues 255–275 (Fig. 1). While these regions represent good targets for vaccine design, short sequences such as these are generally not able to adopt a native, helical conformation when synthesised as peptides^37^. Therefore, we also generated peptide versions which were flanked at both termini by an α-helicity inducing GCN4 sequence (**ØP2** and **IIP4**) as well as an elongated form of the site II sequence (**IIP5**). CD spectra of LCP vaccine candidates correlated well with the computational prediction of secondary structure and LCP constructs that incorporated chimeric or elongated peptides showed α-helical conformation in solution. Both α-helix and β-sheet spectra were observed for LCP constructs bearing native minimal B cell sequence (Supplementary Fig. S16).

Encouragingly, several of the constructs were recognised by their cognate antibody (Fig. 3). Site Ø candidate, **ØLCP2**, was bound by D25 with nanomolar avidity (k_D_ = 66 ± 14 nM) while modest binding was observed for **ØP2** peptide alone (~720 nM) (Fig. 3A). Neither **ØP1** nor **ØLCP1** bound to D25 suggesting that the addition of the GCN4 motif, and associated increased propensity for helix formation, is critical for D25 recognition. Indeed, **ØLCP2** formed a predominantly helical conformation in solution and bound D25 with highest avidity. Similarly, site II presentation was enhanced by the addition of a GCN4 motif, with **IILCP4** bound Motavizumab showing the strongest antibody binding avidity of any vaccine candidate (k_D_ = 3.8 ± 0.9 nM) (Fig. 3B). In comparison, we found that D25 and Motavizumab bound to the full ectodomain of native pre-fusion RSV F (F_Ds-Cav1_) with around 60-fold and 25-fold stronger affinities, respectively (k_D_ = 2.4 ± 00.2 nM and 0.15 ± 00.01nM).

After confirming antibody recognition we evaluated the ability of the LCP candidate to self-assemble into discrete nanoparticles, since antigen particle size has been proposed to play a vital role in vaccine delivery and efficacy *in vivo* ^55^. DLS analysis revealed that **ØLCP2** formed small nanoparticles of around 40 nm diameter, while **ØLCP1** and **IILCP4-7** formed larger particles (100–500 nm) (Table 1). **IILCP3** formed even larger aggregates with a diameter in the micron range. The formation of large particles by **IILCP3** is most likely related to its secondary structure. It is well-recognised that peptides forming β-sheets have a tendency to aggregate^56^. LCPs that formed smaller nanoparticles (e.g. **ØLCP2**, **IILCP4** and **IILCP5**) generally induced higher antibody titres than their larger counterparts (e.g. **IILCP3**). A robust antibody response was observed for most LCP candidates (**ØLCP1, ØLCP2, IILCP4** and **IILCP5**) (Fig. 4A,D). In comparison, a strong response was only observed for CFA-adjuvanted peptide **IIP5** (Fig. 4A,D).

While these findings indicate that the LCP delivery system potentiates immunogenicity, it is essential that the induced antibodies are able to react with the native viral antigen. Persuasive evidence also indicates that the recognition of the pre-fusion form of viral fusion glycoproteins is critical for the generation of protective antibodies^11, 21^. Sera from vaccinated animals were tested for the presence of pre- and post-fusion F specific antibodies together with PRNT assays to determine the neutralising antibody response. Unfortunately, we observed very little reactivity with either the pre- or post-fusion F for any of the peptide or LCP constructs (Fig. 4B,C,E,F). Only sera from F_sol_ immunised animals was able to recognise Fsol, but surprisingly showed very little cross-reactivity to pre-fusion F_Ds-Cav1_. To confirm the ELISA results we also used immunofluorescence to examine reactivity with RSV antigen in infected cells (Supplementary Fig. S17). Again, only sera from F_sol_ immunised animals was able to recognise RSV infected cells, likely due to recognition of post-fusion F.

In accordance with the low level of native F recognition, none of the mouse sera collected following peptide vaccinations was able to neutralise RSV. These finding may reflect the complex nature of the target epitopes which both require quaternary contacts for optimal antibody affinity. Illustrating this point, structural studies have revealed that D25 makes direct contacts with multiple components of the RSV F protein. The D25 heavy chain binds to F1 (residues 196–210) and also interacts with F2 (residues 63, 65, 66 and 68) forming intermolecular hydrogen bonds^10^. The D25 light chain binds to F1 (residues 196–210) but also interacts with residues 70–74 on the neighbouring protomer. Therefore, only including the F1 subunit residues 196–211, might not meet all requirements for success of an applied epitope-focused approach for site Ø. Likewise, although site II availability is less dependent on trimer conformation, co-crystallisation studies revealed that there are additional interactions beyond residues 255–275. The Motavizumab heavy chain also makes an interaction with K465 and the Motavizumab light chain also interacts with N454 of the neighbouring protomer. A potential additional issue associated with peptide-based approaches is that all amino acids within the peptide are potentially available for antibody affinity maturation, whereas in the context of the native protein a component of the peptide may be naturally buried within the protein.

To our knowledge this is the first report of the synthesis of a high affinity peptide mimetic of site Ø. Thus, despite the lack of neutralising response observed from these candidates, D25 reactivity warrants further study. It may be possible that if presented using an appropriate scaffold such peptides could adopt the correct conformation and orientation to further mimic the native F protein. Alternatively, similar mimetics could be used in a prime-boost regime together with stabilised pre-fusion F to direct the host response to site Ø.

The main goal of epitope-focused vaccine design is to exploit the minimal immunogenic fragment of a pathogen that is capable of generating a protective immune response. To this end we successfully synthesised a panel of self-adjuvanting epitope-focused RSV vaccine candidates using SPPS. Neutralising epitopes (antigenic site Ø and II) were incorporated into a LCP delivery system with the aim of generating an immune response capable of blocking the virus at initial phases of infection. Although secondary structure analysis and recognition by cognate antibodies suggested that some of the vaccine candidates were able to correctly present native epitopes in a minimised format, these constructs failed to produce a neutralising response *in vivo*. To our knowledge, this is the first report of incorporation of antigenic site Ø in a peptide-based vaccine, and the first to report a small peptide mimetic of this site with high avidity to the highly neutralising D25 antibody. However, while monoclonal antibodies against the Ø and II epitopes have been shown to provide effective virus neutralisation, our results indicate that peptide presentation of these epitopes in an α-helical conformation is not sufficient to elicit a neutralising response within a murine model.

## Methods

### Design of epitope

The crystal structure of RSV F protein stabilised in the pre-fusion state (PDB code: 4JHW) was evaluated using PyMOL Molecular Graphics System Version 1.3 (Schrödinger, LLC) software and the secondary structures of B cell epitope peptides, **ØP1 (**residues 196–211, KNYIDKQLLPIVNKQS) and **IIP3** (residues 255–275, SELLSLINDMPITNDQKKLMSNN) were extracted. Additional constructs, **ØP2** and **IIP4**, were generated based on the same sequences but included terminal coil-promoting sequences from yeast GCN4 protein^38^. A further elongated peptide **IIP5** was also generated incorporating addition native residues at both termini of sequence **IIP3** (residues 252–283, LTNSELLSLINDMPITNDQKKLMSNNVQIVRQQS). The secondary structure of each peptide construct was analysed by *in silico* prediction tools Porter^39^, PSIPRED^40^ and SCRATCH^41^.

### Synthesis of peptides and vaccine candidates

Peptides (**ØP1** and **ØP2**) were synthesised using microwave-assisted SPPS at 0.2 mmol scale on Rink amide MBHA (0.45 mmol/g) resin in a similar manner as previously published^42, 43^. Briefly, N-Fmoc-protected amino acids (4.2 equiv) were dissolved in 0.5 M HATU/DMF solution (4.0 equiv) and DIPEA (6.2 equiv). Coupling cycle consisted of Fmoc deprotection with 20% of piperidine in DMF (twice, 2 and 5 min, 70 °C), a 1 min DMF flow-wash and coupling pre-activated amino acids (twice, 5 and 10 min) under microwave irradiation condition at 70 °C. Upon completion of peptide synthesis, peptides were acetylated at the N-terminus (acetic anhydride, DIPEA, DMF 0.5:0.5:9, 2 × 10 min, 70 °C). Peptides were cleaved in peptide cleavage cocktail (TFA/H_2_O/TIS (95:2.5:2.5% *v/v/v*)) for 4 h at room temperature. Cold Et_2_O was used to precipitate the cleaved peptides. Peptides were then separated from the resin using aqueous acetonitrile (50% *v/v*) solution. The crude product was purified using preparative high performance liquid chromatography (HPLC) on a C-18 column with a 30–50% solvent B gradient over 60 min (0.1% TFA/H_2_O as solvent A and 90% MeCN/0.1% TFA/H_2_O as solvent B).  Then HPLC analysis was performed using a 0–100% linear gradient of solvent B over 40 min and pure fractions were analysed by Electrospray ionisation-mass spectrometry (ESI-MS) (Supplementary Fig. S1,2).

Peptide **ØP1**. Yield: 27%. Molecular Weight: 1942.3. ESI-MS [M+2H^+^]^2+^ m/z 972.0 (calc. 972.1), [M+3H^+^]^3+^ m/z 648.4 (calc. 648.4), [M+4H^+^]^4+^ m/z 486.4 (calc. 486.5). t_R_= 20.60 min (0–100% solvent B; C18 column), purity ≥95.

Peptide **ØP2**. Yield: 21%. Molecular Weight: 3623.2. ESI-MS [M+3H^+^]^3+^ m/z 1208.9 (calc. 1208.7), [M+4H^+^]^4+^ m/z 906.7 (calc. 906.8), [M+5H^+^]^5+^ m/z 725.6 (calc. 725.6), [M+6H^+^]^6+^ m/z 604.9 (calc. 604.8). t_R_= 26.29 min (0–100% solvent B; C18 column), purity ≥95.

**IIP3**-**5** and PADRE were synthesised on *p*-methylbenzhydrylamine resin (*p*-MBHA) via *t*-butyloxycarbonyl (Boc) chemistry using microwave-assisted SPPS^32, 42^. Briefly, N-Boc-protected amino acids (4.2 equiv) were dissolved in 0.5 M HATU/DMF solution (4.0 equiv) and DIPEA (6.2 equiv). Coupling cycle consisted of a Boc deprotection step with TFA (twice, 1 min, at room temperature), a 1 min DMF flow-wash, followed by coupling of pre-activated amino acids (twice, 5 min and 10 min, under microwave irradiation condition at 70 °C). Upon completion of peptide synthesis, peptides were acetylated at the N-terminus (acetic anhydride, DIPEA, DMF 0.5:0.5:9, 2 × 10 min, 70 °C). PADRE was cleaved using a HF/thiocresol/cresol (90/5/5% v/v) cocktail mixture at -5 °C^44^. Peptides were then precipitated and further purified by HPLC and analysed by analytical HPLC and ESI-MS as described above (Supplementary Fig. S3–6).

Peptide **IIP3**. Yield: 12%. Molecular Weight: 2660.0. ESI-MS [M+2H^+^]^2+^ m/z 1331.3 (calc. 1331.0), [M+3H^+^]^3+^ m/z 887.8 (calc. 887.6), [M+4H^+^]^4+^ m/z 666.1 (calc. 666.0). t_R_= 23.25 min (0–100% solvent B; C18 column), purity ≥95.

Peptide **IIP4**. Yield: 15%. Molecular Weight: 3471.9. ESI-MS [M+2H^+^]^2+^ m/z 1736.7 (calc. 1736.9), [M+3H^+^]^3+^ m/z 1158.1 (calc. 1158.3), [M+4H^+^]^4+^ m/z 868.9 (calc. 868.9), M+5H^+^]^5+^ m/z 695.4 (calc. 695.3). t_R_= 26.06 min (0–100% solvent B; C18 column), purity ≥95.

Peptide **IIP5**. Yield: 20%. Molecular Weight: 3927.5. ESI-MS [M+3H^+^]^3+^ m/z 1310.3 (calc. 1310.1), [M+4H^+^]^4+^ m/z 982.8 (calc. 982.8). t_R_= 25.21 min (0–100% solvent B; C18 column), purity ≥95.

**PADRE**. Yield: 52%. Molecular Weight: 1388.8. ESI-MS [M+2H^+^]^2+^ m/z 695.3 (calc. 695.4), [M+3H^+^]^3+^ m/z 463.8 (calc.463.9). t_R_= 23.88 (0–100% solvent B; C18 column), purity ≥95.

LCP constructs (**ØLCP1** and **ØLCP2**) were synthesised by microwave-assisted SPPS at 0.2 mmol scale on Rink amide MBHA (0.45 mmol/g) resin. Sixteen-carbon lipoamino acid (C16; 2-((1-(4,4-Dimethyl-2,6-dioxocyclohexylidene)ethyl)amino)hexadecanoic acid (Dde-C16-OH) was synthesised as previously described^45, 46^ (Supplementary Fig. S7). The Dde-C16 (4.2 equiv.) was activated in 0.5 M HATU/DMF solution (4.0 equiv) and DIPEA (6.2 equiv) for 5–10 min before coupling. Lipoamino acids were coupled twice for at least 1 h at room temperature. Dde was deprotected by treatments with 2% (v/v) hydrazine hydrate in DMF (10 × 15 min). It was monitored by the absorbance at 290 nm until no changes was observed. Then the linker sequence (two serine and a Fmoc-Lys(Mtt)-OH) and T helper epitope (PADRE), AKFVAAWTLKAAA, were synthesised using microwave-assisted SPPS. Fmoc deprotection was performed using 20% of piperidine in DMF (twice, 2 and 5 min, 70 °C). N-terminus amine group was acetylated in the same manner as described above. At the branching point (Lys-(Mtt)-OH), the ε-Mtt protecting group was removed using cocktail of 1% TFA and 5% TIPS in DCM (20 × 5 min) followed by washing with DCM and DMF. Then the B cell epitope was synthesised and acetylated at the N-terminus. The product was purified using a preparative HPLC on a C-4 column with a 55–65% solvent B gradient over 60 minutes. The fractions contained pure product were collected and analysed by analytical HPLC and ESI-MS as above (Supplementary Fig. S8,9).

**ØLCP1**. Yield: 16%. Molecular Weight: 4123.1. ESI-MS [M+3H^+^]^3+^ m/z 1375.3 (calc. 1375.3), [M+4H^+^]^4+^ m/z 1031.6 (calc. 1031.7), [M+5H^+^]^5+^ m/z 825.7 (calc. 825.6), [M+6H^+^]^6+^ m/z 688.0 (calc. 688.1). t_R_= 31.85 min (0–100% solvent B; C4 column), purity ≥95.

**ØLCP2**. Yield: 14%. Molecular Weight: 5804.1. ESI-MS [M+3H^+^]^3+^ m/z 1935.2 (calc. 1935.7), [M+4H^+^]^4+^ m/z 1452.4 (calc. 1425.0), [M+5H^+^]^5+^ m/z 1162.3 (calc. 1161.8), [M+6H^+^]^6+^ m/z 968.3 (calc. 968.3), [M+7H^+^]^7+^ m/z 830.0 (calc. 830.1). t_R_= 31.44 min (0–100% solvent B; C4 column), purity ≥95.

LCP constructs including B-cell epitope from antigenic site II (**IILCP3**-**7**) were synthesised on *p*-methylbenzhydrylamine resin (*p*-MBHA) via *t*-butyloxycarbonyl (Boc) chemistry using manual and microwave-assisted SPPS resin in a similar manner as previously published^32, 42^. Dde-C16 was activated in 0.5 M HATU/DMF solution (4.0 equiv) and DIPEA (6.2 equiv). Dde was deprotected by several 15 min treatments with 2% (v/v) hydrazine hydrate in DMF (20 × 15min). Complete Dde-removal was performed as above. Then the linker sequence (two serine and a Boc-Lys-Fmoc) and T helper epitope (PADRE), AKFVAAWTLKAAA, were synthesised using microwave-assisted SPPS and N-terminus amine group was acetylated. At the branching point, Fmoc protecting group removed using 20% of piperidine in DMF (twice, 2 and 5 min, 70 °C) followed by washing with DMF. Following this the B cell epitope was synthesised as above and acetylated at the N-terminus. For **IILCP6** and **IILCP7** two extra copies of lipoamino acid were coupled at the N-terminus of the synthesised B cell epitope and acetylated at the N-terminus. The product was purified using a preparative HPLC on a C-4 column with a gradient of solvent B (**IILCP3-5**, 55-85%; **IILCP6** and **IILCP7**, 80–100%) over 60 minutes. The fractions contained pure product were collected and analysed by analytical HPLC and ESI-MS (Supplementary Fig. S10–14).

Peptide **IILCP3**. Yield: 12%. Molecular Weight: 4840.9. ESI-MS [M+3H^+^]^3+^ m/z 1614.8 (calc. 1614.6), [M+4H^+^]^4+^ m/z 1210.9 (calc. 1211.2), [M+5H^+^]^5+^ m/z 969.0 (calc. 969.1), [M+6H^+^]^6+^ m/z 807.7 (calc. 807.8), t_R_= 27.73 min (0–100% solvent B; C4 column), purity ≥95.

Peptide **IILCP4**. Yield: 16%. Molecular Weight: 5652.8. ESI-MS [M+4H^+^]^4+^ m/z 1414.1 (calc. 1414.2), [M+5H^+^]^5+^ m/z 1131.5 (calc. 1131.5), [M+6H^+^]^6+^ m/z 943.0 (calc. 943.1), [M+7H^+^]^7+^ m/z 808.5 (calc. 808.5), [M+8H^+^]^8+^ m/z 707.5 (calc. 707.6). t_R_= 32.61 min (0–100% solvent B; C4 column), purity ≥95.

Peptide **IILCP5**. Yield: 23%. Molecular Weight: 6108.3. ESI-MS [M+5H^+^]^5+^ m/z 1222.7 (calc. 1222.6), [M+6H^+^]^6+^ m/z 1019.0 (calc. 1019.0), [M+7H^+^]^7+^ m/z 873.7 (calc. 873.6). t_R_= 31.86 min (0–100% solvent B; C4 column), purity ≥95.

Peptide **IILCP6**. Yield: 13%. Molecular Weight: 6159.6. ESI-MS [M+5H^+^]^5+^ m/z 1233.0 (calc. 1232.9), [M+6H^+^]^6+^ m/z 1027.6 (calc. 1207.6). t_R_= 32.82 min (50–100% solvent C; C4 column), purity ≥95.

Peptide **IILCP7**. Yield: 10%. Molecular Weight: 6615.2. ESI-MS [M+5H^+^]^5+^ m/z 1324.3 (calc. 1324.0), [M+6H^+^]^6+^ m/z 1103.5 (calc. 1103.5). t_R_= 32.88 min (50–100% solvent C; C4 column), purity ≥95.

### Particle Size Analysis using Dynamic Light Scattering

Peptides were dissolved in 1% DMSO in phosphate buffered saline (PBS) to a concentration of 600 µg/ml. Particle size was measured using a Zetasizer Nano ZP instrument (Malvern Instrument, UK) via M3-PALS technique with a 173° backscattering angle, at 25 °C. Measurement was performed in five replicates.

### Peptide secondary structure analysis by spectropolarimetry

Conformational studies of constructs were performed using a J-710 spectropolarimeter (Jasco Corporation, Japan). Samples with 600 µg/ml concentration were transferred into a low volume quartz cuvette with 0.1 cm path length. CD spectra were analysed between 250 nm and 190 nm wavelengths. Measurements were performed in triplicate (reported as an average of readings). Spectral results are reported as mean residual ellipticity (θ) versus wavelength (nm).

### Cells and viruses

Vero cells were maintained in Opti-MEM media (Invitrogen) supplemented with 3% fetal bovine serum (FBS). CHO cells were maintained in CD-CHO (Invitrogen) supplemented with 8mM Glutamax. A549 cells were grown in DMEM/F12 (Invitrogen) with 10% FBS. Vero and A549 cell lines were grown at 37 °C in a humidified incubator supplemented with 5% CO_2_. CHO cells were grown in suspension phase on a rotary shaker at 120 rpm, within a 37 °C in a humidified incubator supplemented with 7.5% CO_2_. RSV (A2) was grown in A549 cells. RSV titres were determined by serial dilution on Vero cells using an immunoplaque detection method as previously described^47^.

### Cloning of RSV fusion proteins

A soluble recombinant version of RSV F (F_sol_) lacking the transmembrane and cytoplasmic domains (amino acids 1–516) was cloned into pIRES2-EGFP expression vector (Clontech^TM^). A C-terminal 4Gly linker and 6His tag were added to facilitate purification. A stabilised pre-fusion conformation of soluble RSV F (RSV F_Ds-Cav1_) was also produced based on the method utilised by McLellan *et al*.^21^. Briefly, two cavity filling mutations (S190F, V207L) and an additional disulphide bridge S155C-S290C were incorporated into RSV F and the foldon trimerisation sequence was incorporated at the C-terminus.

### Recombinant antibody cloning

The variable regions of the D25 and Motavizumab antibodies were cloned into plasmids encoding the human constant heavy and light chain backbone sequences as previously described^48^. Briefly, variable region sequences were obtained from the PDB database and codon-optimised for CHO cell expression by GeneArt GeneOptimizer (ThermoFisher Scientific), with complementary sequences added to the 5′ and 3′ ends to facilitate In Fusion^TM^ cloning (Clontech) into mouse and human constant domain expression scaffolds using mAbXpress heavy and kappa chain vectors, kindly provided by Dr Martina Jones, AIBN, University of Queensland. These constructs were synthesised as a gBlock (Integrated DNA Technologies) and cloned into the mAbXpress vectors linearised by SacI (New England Biolabs).

### Recombinant Protein expression

CHO cells were transfected with pIRES2-EGFP F_sol_ or F_Ds-Cav1_ at a ratio of 2 µg DNA and 8 µM linear polyethylenimine per 1 × 10^6^ cells. Cells were incubated with transfection reagent for 4 h without shaking at 37 °C, 7.5% CO_2_. Cells were then pelleted by centrifugation at 200 × g for 10 min and resuspended in CD-CHO (Gibco^TM^), with 8 mM GlutaMAX^TM^ (Gibco^TM^), 7.5% CHO CD EfficientFeed^TM^ A Liquid Nutrient Supplement (Gibco^TM^), 7.5% CHO CD EfficientFeed^TM^ B Liquid Nutrient Supplement (Gibco^TM^) and 1x Penicillin-Streptomycin (Gibco^TM^). Cells were then incubated on a rotary shaker at 37 °C, 7.5% CO_2_ for 7 days before harvesting.

### Recombinant Protein Purification

RSV F_sol_ was purified by nickel affinity chromatography. Briefly, CHO expression cultures were centrifuged to pellet cell debris at 4000 × g for 10 min and supernatant was filtered. Supernatant was added to Ni Sepharose excel (GE^TM^) that was pre-equilibrated with 50 mM HEPES, pH 7.5, 300 mM NaCl, 10 mM imidazole and incubated for 30 min at 4 °C. Bound resin was washed extensively with 50 mM HEPES, pH 7.5, 300 mM NaCl, 40 mM imidazole and protein eluted with 50 mM HEPES, pH 7.5, 300 mM NaCl, 300 mM imidazole. Purification of RSV F_sol_ was confirmed by sodium dodecyl sulfate polyacrylamide gel electrophoresis (SDS-PAGE) and Western blot analysis.

### Recombinant antibody purification

DNA encoding Motavizumab or D25 antibody constructs was transfected into CHO cells with a 50:50 ratio of heavy:light chain plasmid and harvested after seven days as described above. Antibody was purified with a 1 ml Protein-A HiTrap column (GE^TM^) by FPLC (Akta). The bound sample was washed with PBS, eluted with 0.1 M glycine pH 2.7 and back neutralised with a Tris buffer. Purified antibody then was concentrated and exchanged into PBS using Centrifugal Filtration (Merck Amicon Ultra). Antibody concentration was determined by Pierce BCA Assay (ThermoFisher Scientific) and protein purity confirmed by SDS-PAGE analysis.

### Immunisation study

A total of 70 female C57BL/6 mice (6–8 weeks old) were purchased from the University of Queensland Biological Resources (UQBR), and housed in the AIBN animal house facility. All experimental procedures performed in this study were carried out in accordance with National Health & Medical Research Council guidelines and approved by The University of Queensland Animal Ethics Committee. Mice (n=5 per group) were injected subcutaneously at the tail base on day 0, 21, 28, 35 (Supplementary, Fig S15) with 30 µg of each construct in phosphate-buffered saline (PBS). 1% DMSO was included for LCP constructs to aid solubility. The negative control group were administered with 50 μl of PBS. Control groups included peptides (**ØP1,2 and IIP3–5**) physically mixed with PADRE (1:1) and emulsified in a total volume of 50 μl of complete Freund’s adjuvant (CFA)-PBS (1:1) for the first immunisation and PADRE-peptide mixture in PBS for subsequent immunisations. The soluble form of RSV F lacking transmembrane and cytoplasmic domain (F_sol_) also was used as a positive control and mice were immunised with 7.5 µg/mouse of F_sol_ (emulsified in CFA for the first immunisation).

### Serum collection

Blood was collected via the tail artery from naïve mice and before each boost. Mice were euthanised on day 44 post-immunisation using carbon dioxide (CO_2_) gas and blood was collected via cardiac puncture. Blood was allowed to clot at 4 °C overnight. Serum was collected after centrifugation for 10 min at 1000 × g and stored at −20 °C.

### Determination of IgG antibody titres by ELISA

An indirect enzyme-linked immunosorbent assay (ELISA) was used to determine the IgG antibody titre. Peptide antigens were used to coat 96-well plates (TITERTEK^®^) at a concentration of 5 μg/ml in 20 mM sodium carbonate buffer (pH 9.6), overnight at 4 °C. Non-reactive sites within wells were blocked using 5% Milk Diluent/Blocking Solution Concentrate (KPL, Inc.) in 0.05% Tween 20/PBS for 30 min at room temperature. Plates were washed three times with water. Serial titrations of serum in blocking solution were added and plates were incubated at 37 °C for 1 h. After washing as above, horseradish peroxidase (POD) conjugated goat anti-mouse IgG (eBiosciences) (1:1000 in blocking solution, 50 µl/well) was added and plates incubated at 37 °C for 1 h. Plates were washed as above and secondary antibody binding was detected with the addition of 3,3′,5,5′-Tetramethylbenzidine (TMB) reagent (Thermo Fisher Scientific) at 50 µl/well. Reactions were stopped with 2M H_2_SO_4_ (25 µl/well) and absorbance was read at 450 nm on a SpectraMax*®* 190 (Molecular Devices) microplate reader.

Reactivity of sera against F_sol_ as an antigen was performed as above. Pre-fusion specific reactivity was determined using F_Ds-Cav1_ (RSV F stabilised in pre-fusion conformation) with the following changes. Capture ELISA was performed by adding antigen-specific antibody (either human D25 or human Motavizumab, 2 µg/ml) and ELISA plate (Nunc MaxiSorp®) was incubated overnight at 4°C. CHO supernatant containing F_Ds-Cav1_ was added (50 µl/well) and then plate incubated at 37 °C for 1 h. After washing, serial dilutions of mouse sera were added and incubated at 37 °C for 1 h. All other steps were performed as described above.

### Plaque assay (viral titration)

Vero cells were seeded in 96-well tissue culture plates (Nunc®) at 4 × 10^4^ cells/well in Opti-MEM supplemented with 3% FBS and 100 U penicillin with 100 μg/ml streptomycin. After overnight growth to allow a confluent monolayer, serial dilutions of virus stock were prepared in Opti-MEM (2% FBS and 100 U penicillin with 100 μg/ml streptomycin) and incubated at 37 °C, 5% CO_2_ for 1 h. Cell monolayers were inoculated with 50 μL of virus and incubated. After 1 h, the inoculum was replaced with overlay medium (Medium 199 with 3% methylcellulose, 2% FBS, 100 U penicillin with 100 μg/ml streptomycin) and incubated at 37 °C for 72 h. Overlay medium was aspirated and cells were fixed (80% acetone) and immunostained with a recombinant anti-RSV monoclonal antibody followed by IRDye® 800CW goat anti-mouse IgG, LI-COR Biosciences (1:2500). Plaques were read using Odyssey® Imager (LI-COR Biosciences) and titre was recorded as PFU/ml.

### RSV Plaque reduction neutralisation test

PRNT assays were performed as previously described^47^. Briefly, pooled Sera was heat inactivated at 56 °C for 30 min. Dilutions of serum were combined with 50 PFU of RSV in serum free Opti-MEM and incubated at 37 °C for 1 h before applying to confluent monolayers of Vero cells in TC96-well plates. Cells were incubated with the serum-virus mixture at 37 °C for 1 h. Inoculum was aspirated and overlay medium was added and cells were incubated at 37 °C for 72 h. Cells were fixed and immunostained as for plaque assay as described above.

### RSV Immunofluorescence staining

Vero cells were seeded in 96-well tissue culture plates (Nunc®) at 4 × 10^4^ per well in Opti-MEM supplemented with 3% FBS and incubated at 37 °C for 24 h. Then cells were treated with Opti-MEM SFM as mock infection or infected with RSV A2 for 48 h. Cells were washed in PBS and fixed with 4% paraformaldehyde for 10 min at room temperature. The cells were incubated with mouse sera serially diluted 3-fold, starting at a 1:20 initial dilution at 37 °C for 1 h. Plates were washed with 0.05% Tween 20/PBS three times and incubated with secondary antibodies (Alexa Fluor 555-conjugated donkey anti-mouse IgG, (Molecular Probes™, 1:2000) and incubated at 37 °C for 1 h. HOECHST stain (Hoechst 33342, Invitrogen) was also added at this stage (1:2500). After being washed with 0.05% Tween 20/PBS, rabbit anti-RSV F polyclonal antibody was added (1:500) and incubated at 37 °C for 1 h. Lastly, FITC-conjugated donkey anti-rabbit IgG (Invitrogen) was added (37 °C for 1 h). Then plate was visualised with Zeiss LSM 710 inverted confocal microscope.

## Electronic supplementary material

Supplementary Information
