# Supplementary material for: Induction of high titred, non-neutralising antibodies by self-adjuvanting peptide epitopes derived from the respiratory syncytial virus fusion protein
Source: Sci Rep. 2017 Sep 11;7:11130. doi: 10.1038/s41598-017-10415-w (PMC5593926; doi:10.1038/s41598-017-10415-w)
Supplement: Supplementary file 1 — Supplementary Information [file 41598_2017_10415_MOESM1_ESM.pdf]

Supplementary Information

Induction of high titred, non-neutralising antibodies by self-adjuvanting peptide epitopes derived from the respiratory syncytial virus fusion protein

Authors: Noushin Jaberolansar[1], Keith Chappell[1, 2], Daniel Watterson[1, 2], Imogen M Bermingham[1], Istvan Toth[1,2,3], Paul R Young[1,2], Mariusz Skwarczynski*[1]

Affiliations:

[1] School of Chemistry and Molecular Biosciences, The University of Queensland, St Lucia, Queensland, Australia

[2] Australian Infectious Diseases Research Centre, The University of Queensland, St Lucia, Queensland, Australia

[3] School of Pharmacy, The University of Queensland, Woolloongabba, Queensland, Australia

*To whom correspondence should be addressed:

K.J.C (k.chappell@uq.edu.au), D.W. (d.watterson@uq.edu.au) and M. S. (m.skwarczynski@uq.edu.au)

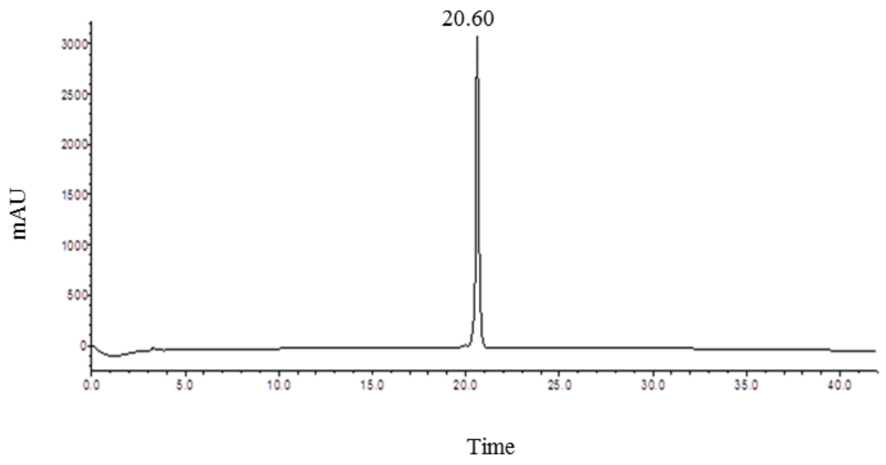

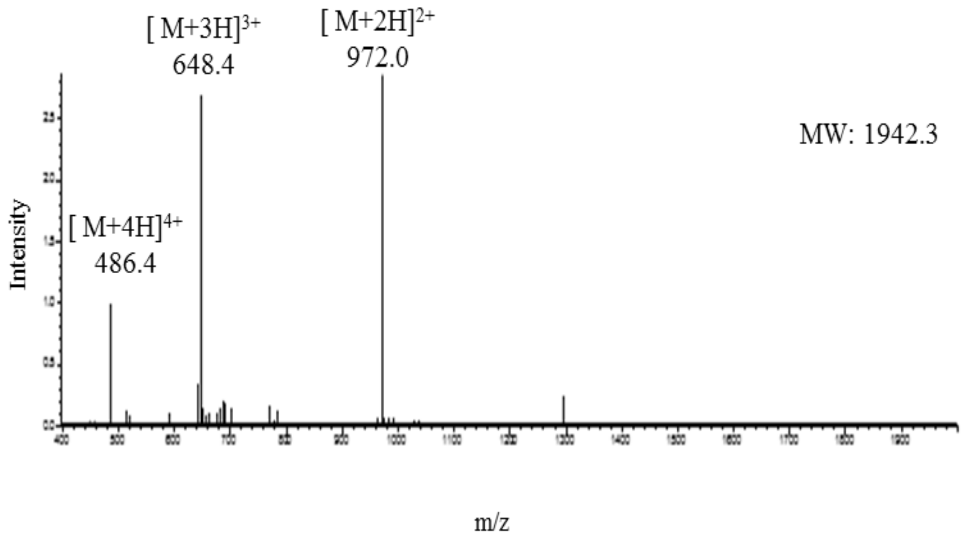

Figure S1 Peptide **ØP1**. Yield: 27%. Molecular Weight: 1942.3. ESI-MS $[M+2H^{+}]^{2+}$ m/z 972.0 (calc. 972.1), $[M+3H^{+}]^{3+}$ m/z 648.4 (calc. 648.4), $[M+4H^{+}]^{4+}$ m/z 486.4 (calc. 486.5). t_R= 20.60 min (0-100% solvent B; C18 column), purity ≥95.

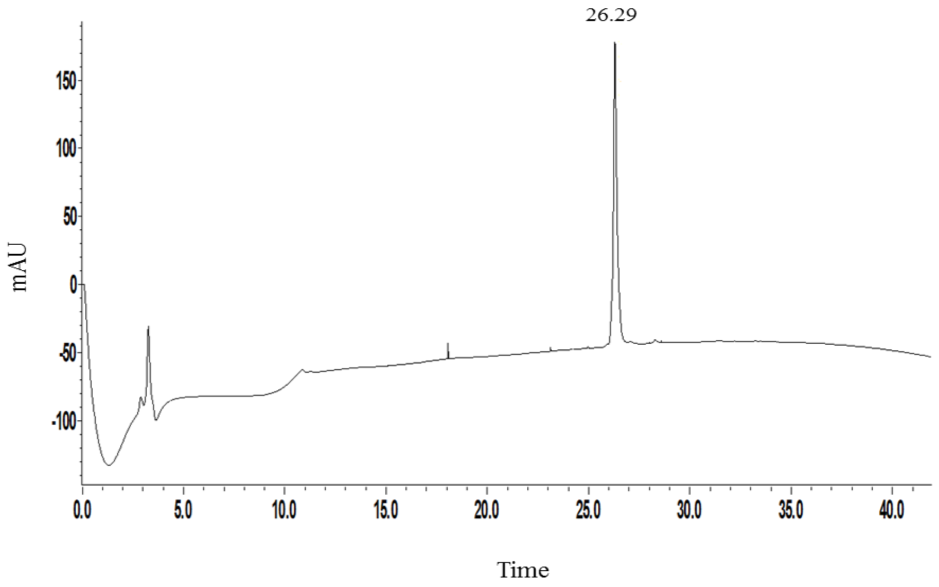

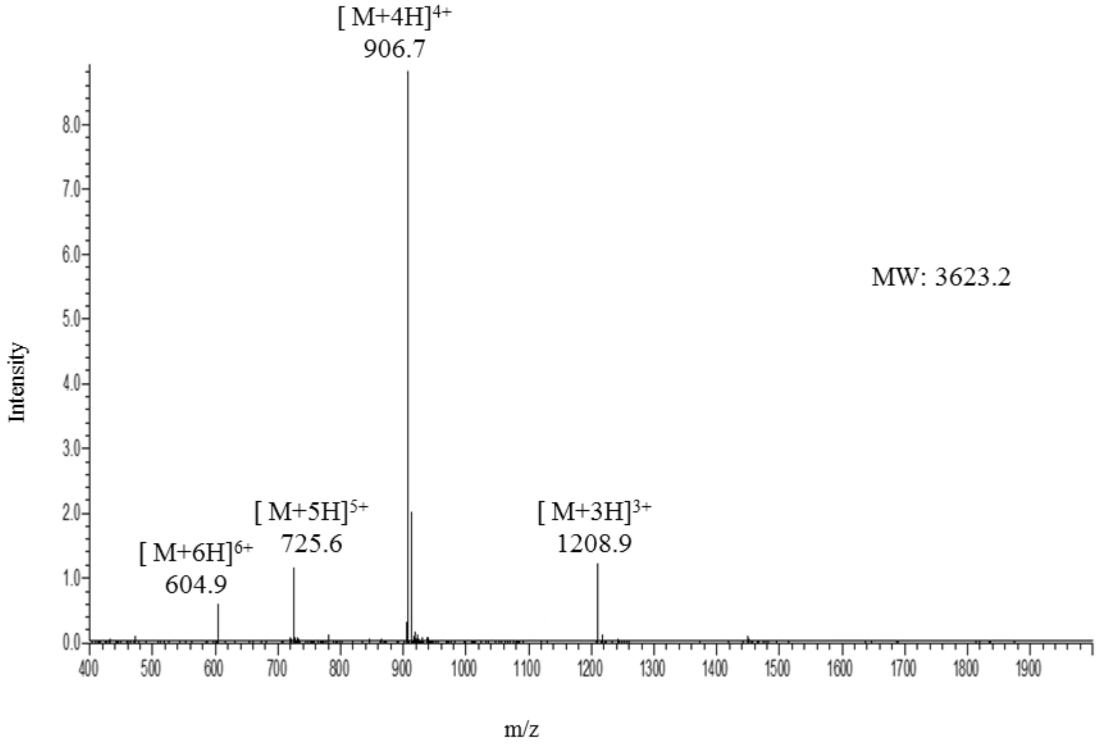

Figure S2 Peptide **ØP2**. Yield: 21%. Molecular Weight: 3623.2. ESI-MS $[M+3H^+]^{3+}$ m/z 1208.9 (calc. 1208.7), $[M+4H^+]^{4+}$ m/z 906.7 (calc. 906.8), $[M+5H^+]^{5+}$ m/z 725.6 (calc. 725.6), $[M+6H^+]^{6+}$ m/z 604.9 (calc. 604.8). t_R= 26.29 min (0-100% solvent B; C18 column), purity ≥95.

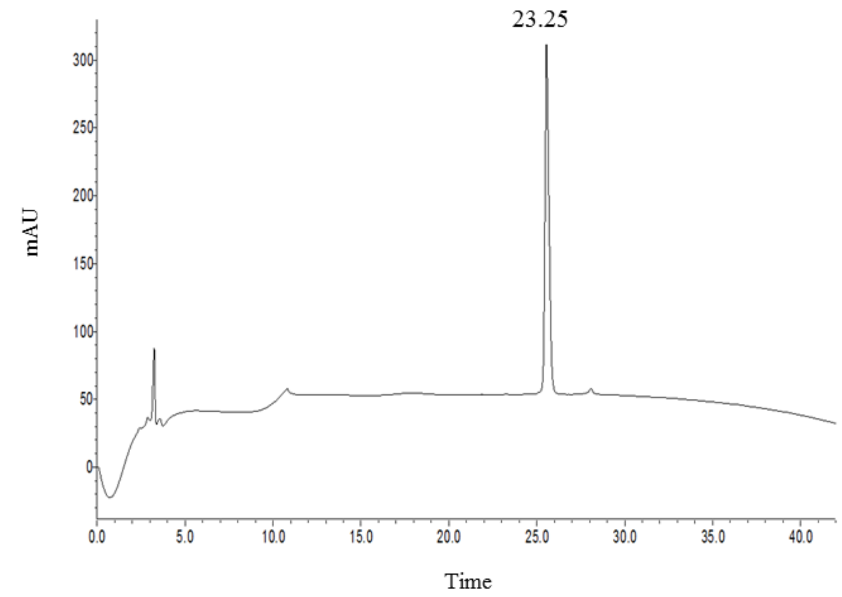

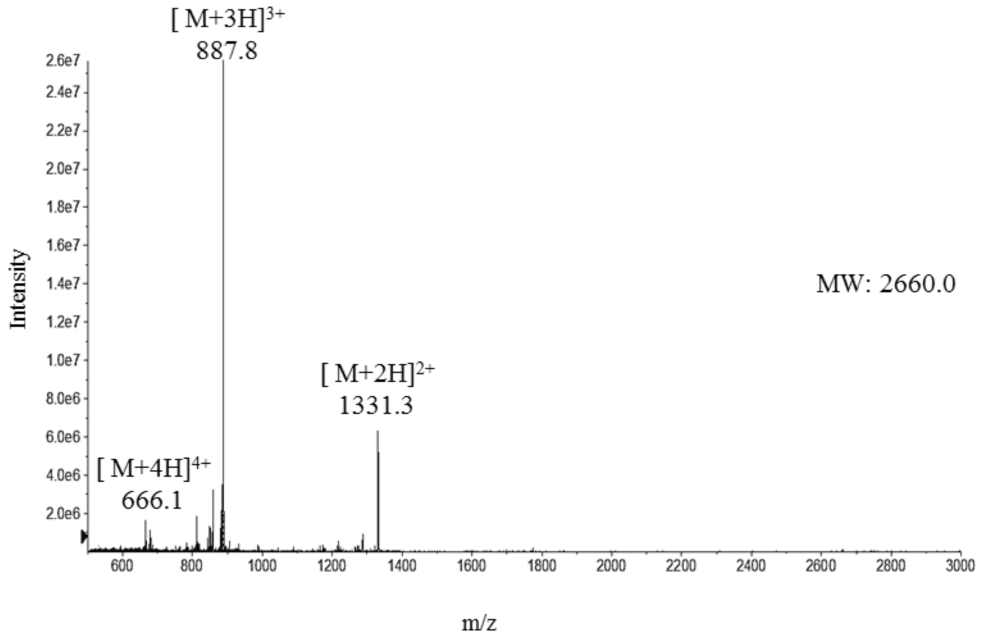

Figure S3 Peptide **IIP3**. Yield: 12%. Molecular Weight: 2660.0. ESI-MS $[M+2H^{+}]^{2+}$ m/z 1331.3 (calc. 1331.0), $[M+3H^{+}]^{3+}$ m/z 887.8 (calc. 887.6), $[M+4H^{+}]^{4+}$ m/z 666.1 (calc. 666.0). t_R= 23.25 min (0-100% solvent B; C18 column), purity ≥95.

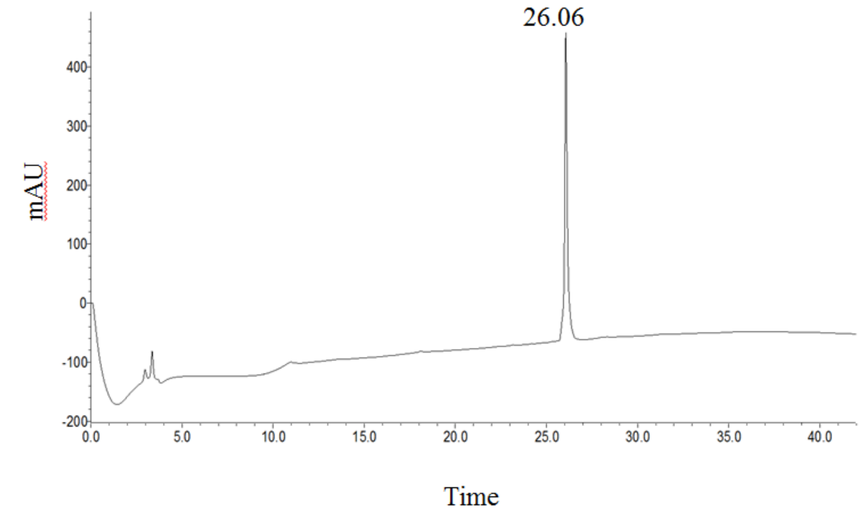

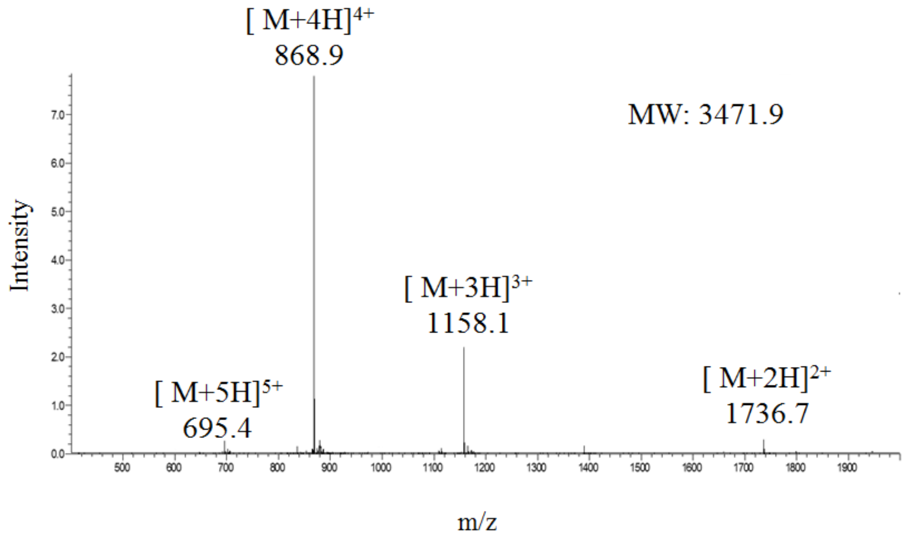

Figure S4 Peptide **IIP4**. Yield: 15%. Molecular Weight: 3471.9. ESI-MS $[M+2H^{+}]^{2+}$ m/z 1736.7 (calc. 1736.9), $[M+3H^{+}]^{3+}$ m/z 1158.1 (calc. 1158.3), $[M+4H^{+}]^{4+}$ m/z 868.9 (calc. 868.9), $M+5H^{+}]^{5+}$ m/z 695.4 (calc. 695.3). t_R= 26.06 min (0-100% solvent B; C18 column), purity ≥95.

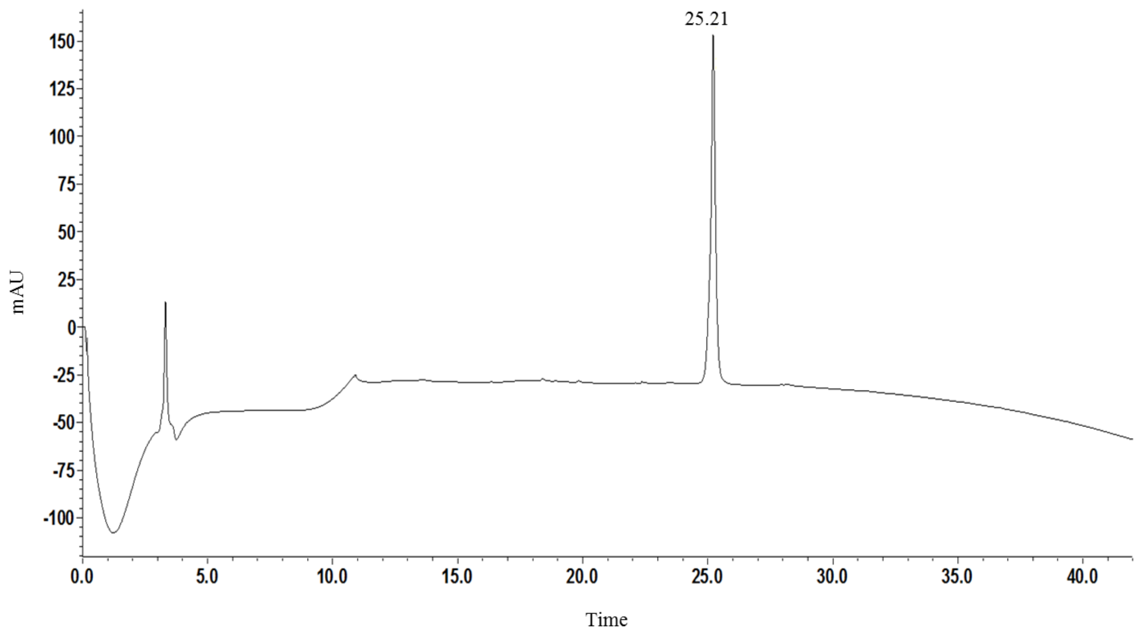

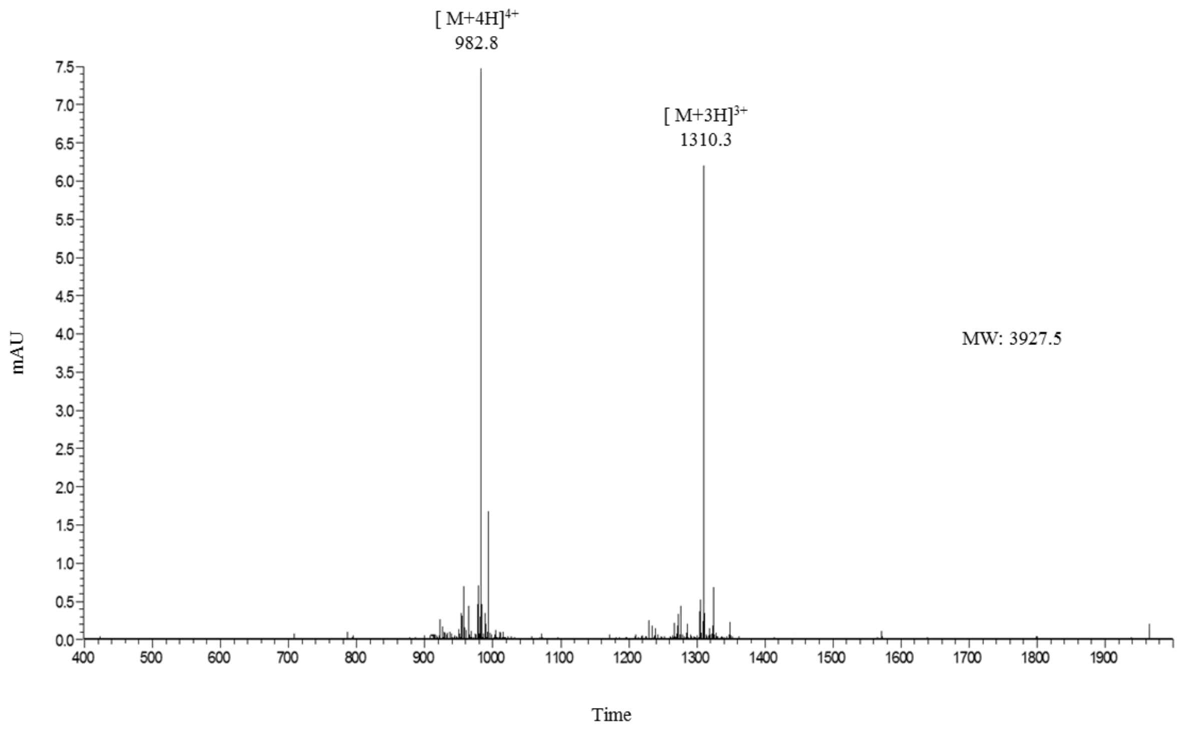

Figure S5 Peptide **IIP5**. Yield: 20%. Molecular Weight: 3927.5. ESI-MS $[M+3H^{+}]^{3+}$ m/z 1310.3 (calc. 1310.1), $[M+4H^{+}]^{4+}$ m/z 982.8 (calc. 982.8). t_R= 25.21 min (0-100% solvent B; C18 column), purity ≥95.

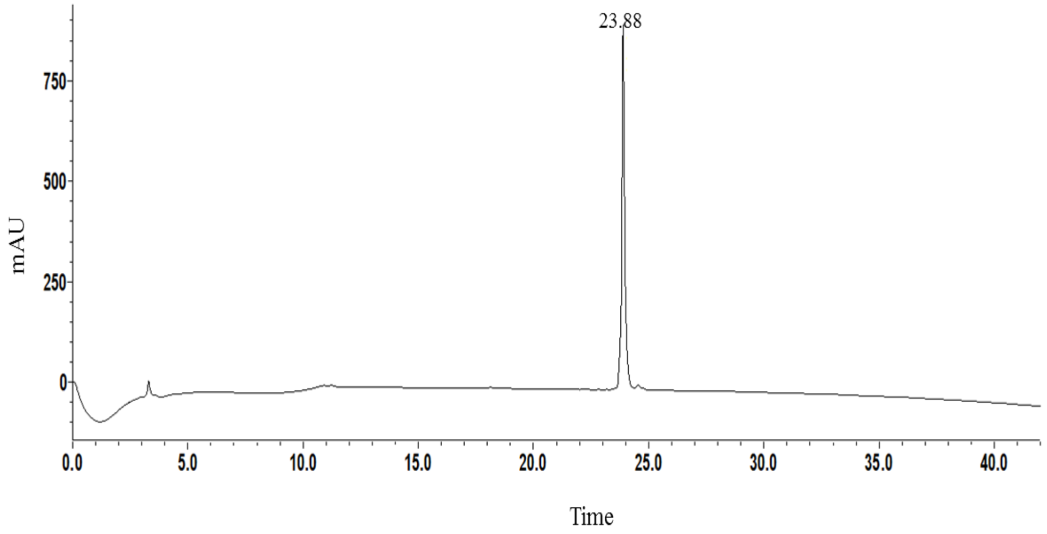

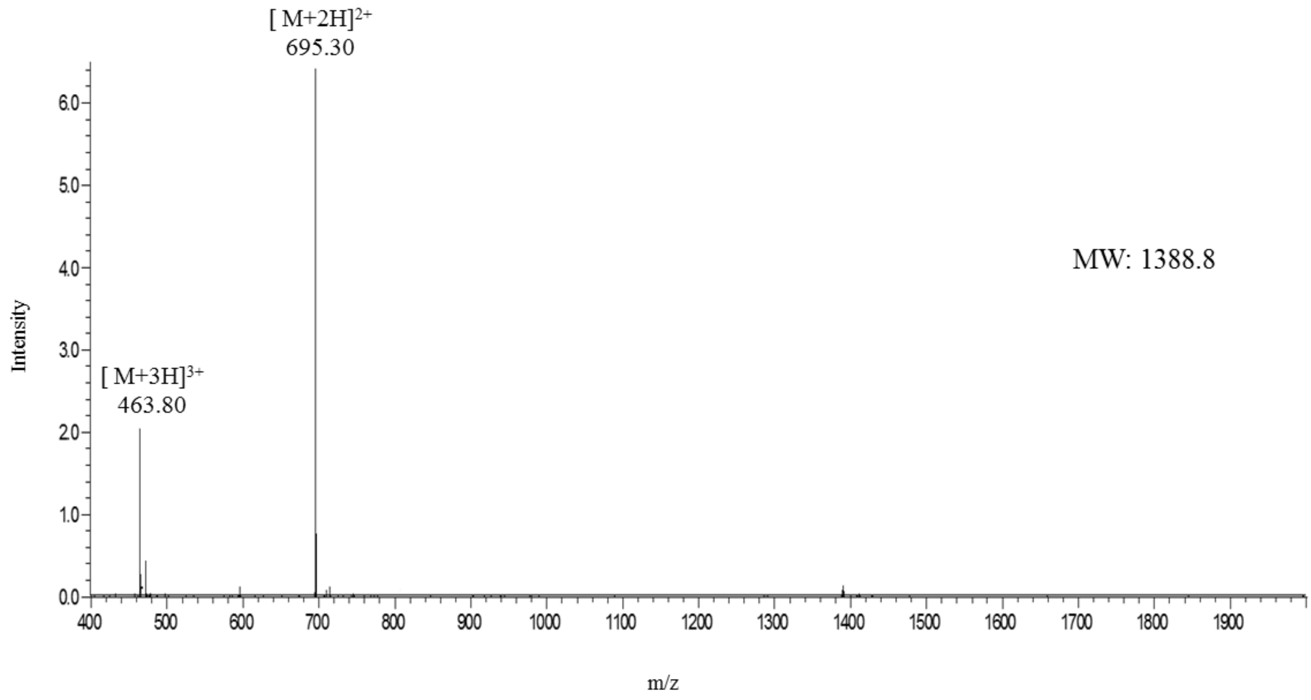

Figure S6 PADRE. Yield: 52%. Molecular Weight: 1388.8. ESI-MS $[M+2H^{+}]^{2+}$ m/z 695.3 (calc. 695.4), $[M+3H^{+}]^{3+}$ m/z 463.8 (calc.463.9). t_R= 23.88 (0-100% solvent B; C18 column), purity ≥95.

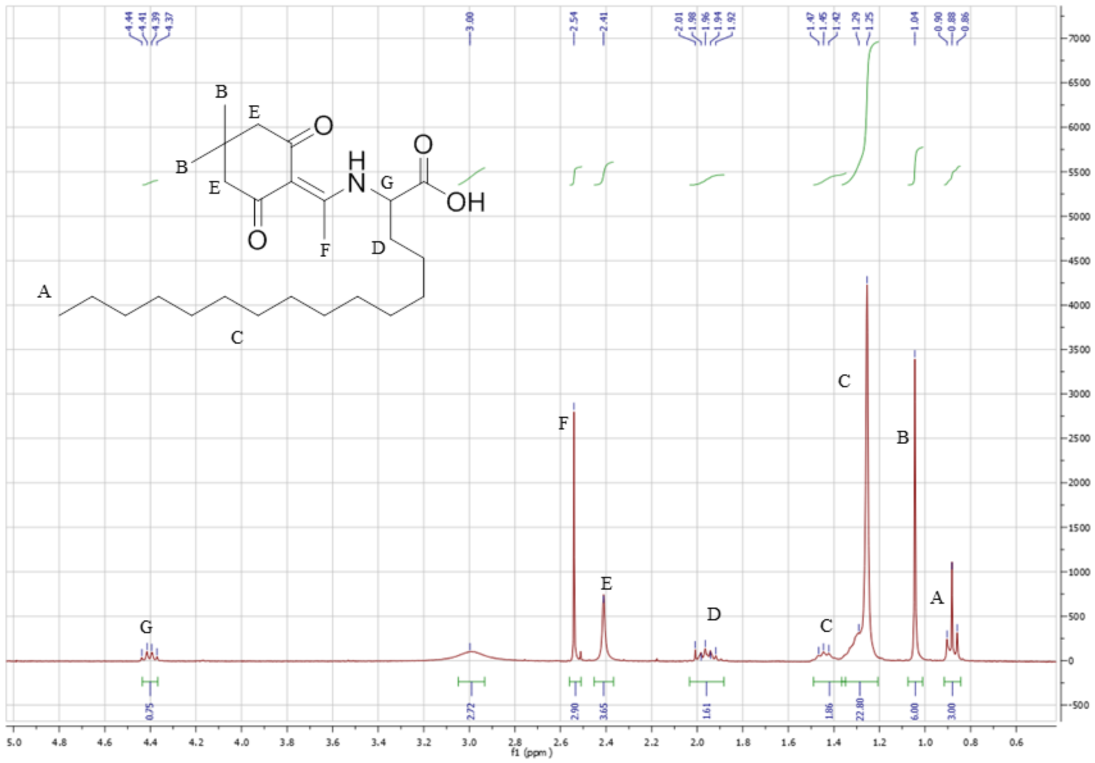

Figure S7 Dde-C16 ^{1}H NMR (500 MHz, $CDCl_3$): 0.86 (t, J=6.9 Hz, 3H, CH_3), 1.04 (s, 6H, CH_3), 1.25-1.29 (m, 22H, CH_2), 1.42-1.47 (m, 2H, CH_2), 1.92-2.01 (m, 2H, CH2), 2.41 (s, 4H, CH_2), 2.54 (s, 3H, CH_3), 3.00(q, J=6.6 Hz, 2H, CH), 4.37-4.44 (s, 1H, NH).

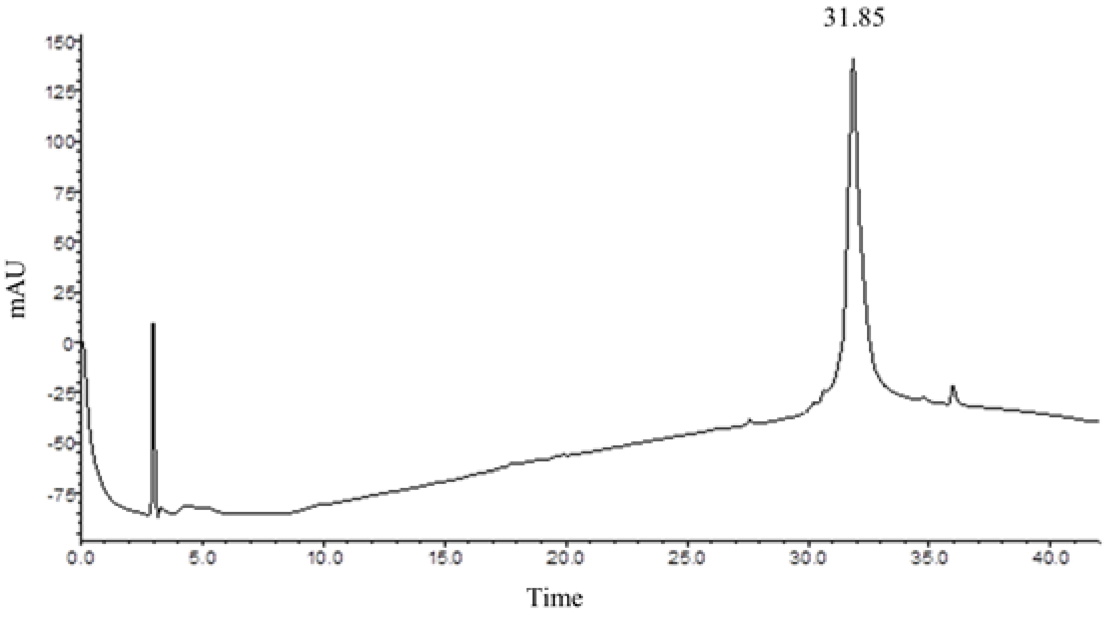

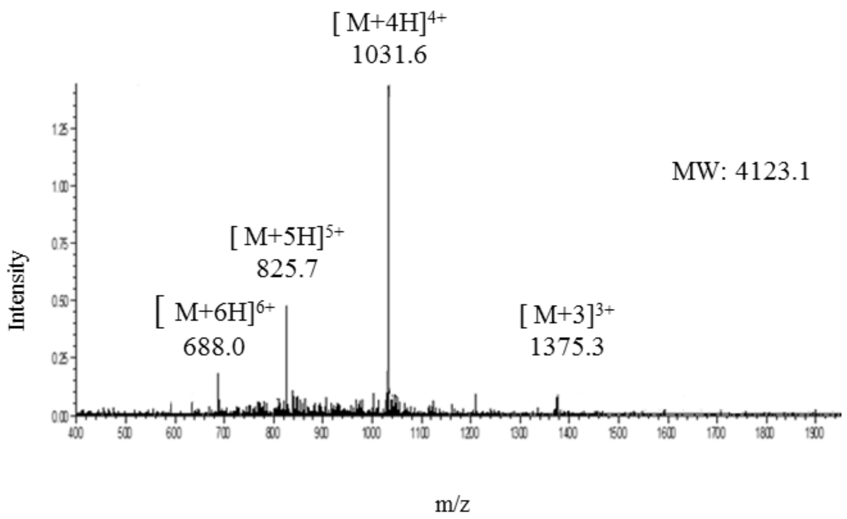

Figure S8 ØLCP1. Yield: 16%. Molecular Weight: 4123.1. ESI-MS $[M+3H^{+}]^{3+}$ m/z 1375.3 (calc. 1375.3), $[M+4H^{+}]^{4+}$ m/z 1031.6 (calc. 1031.7), $[M+5H^{+}]^{5+}$ m/z 825.7 (calc. 825.6), $[M+6H^{+}]^{6+}$ m/z 688.0 (calc. 688.1). t_R= 31.85 min (0-100% solvent B; C4 column), purity ≥95.

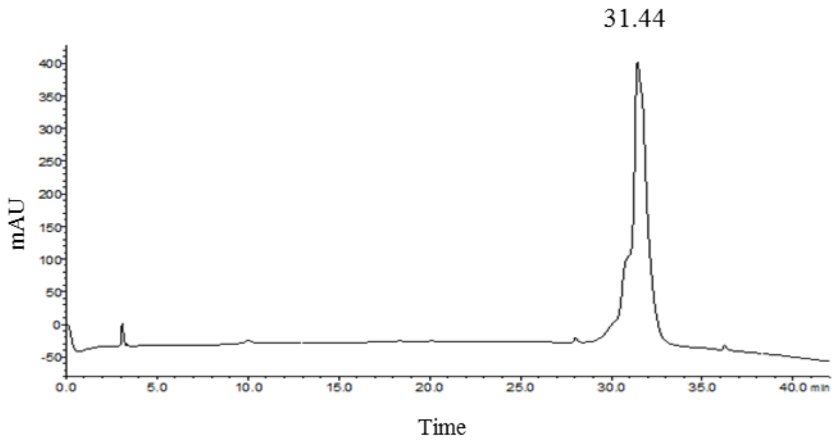

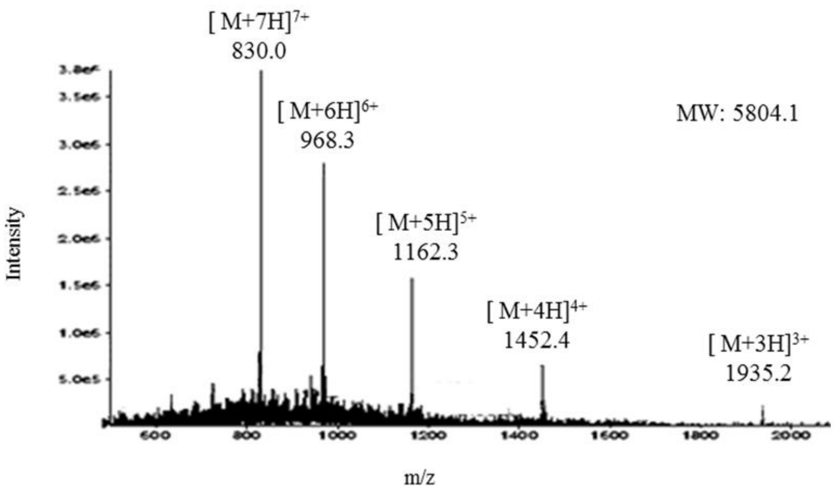

Figure S9 ØLCP2. Yield: 14%. Molecular Weight: 5804.1. ESI-MS $[M+3H^+]^{3+}$ m/z 1935.2 (calc. 1935.7), $[M+4H^+]^{4+}$ m/z 1452.4 (calc. 1425.0), $[M+5H^+]^{5+}$ m/z 1162.3 (calc. 1161.8), $[M+6H^+]^{6+}$ m/z 968.3 (calc. 968.3), $[M+7H^+]^{7+}$ m/z 830.0 (calc. 830.1). t_R= 31.44 min (0-100% solvent B; C4 column), purity ≥95.

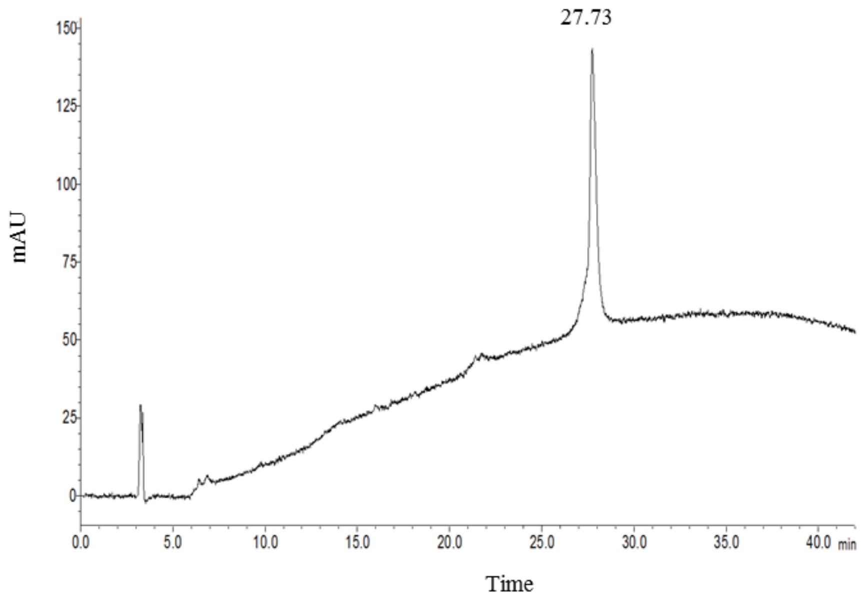

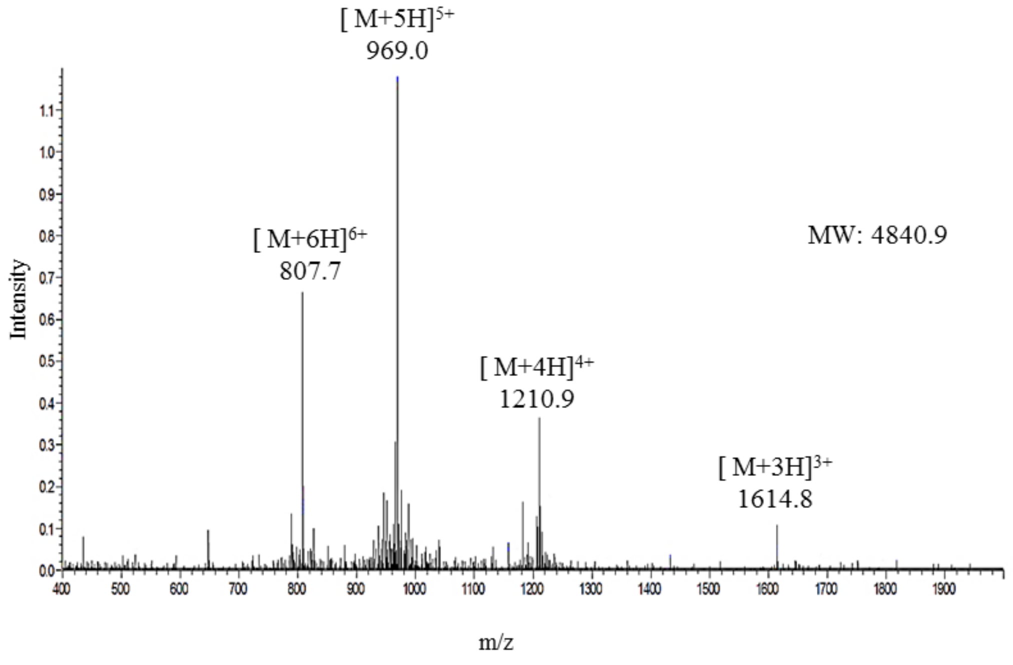

Figure S10 IILCP3. Yield: 12%. Molecular Weight: 4840.9. ESI-MS $[M+3H^+]^{3+}$ m/z 1614.8 (calc. 1614.6), $[M+4H^+]^{4+}$ m/z 1210.9 (calc. 1211.2), $[M+5H^+]^{5+}$ m/z 969.0 (calc. 969.1), $[M+6H^+]^{6+}$ m/z 807.7 (calc. 807.8), t_R= 27.73 min (0-100% solvent B; C4 column), purity ≥95.

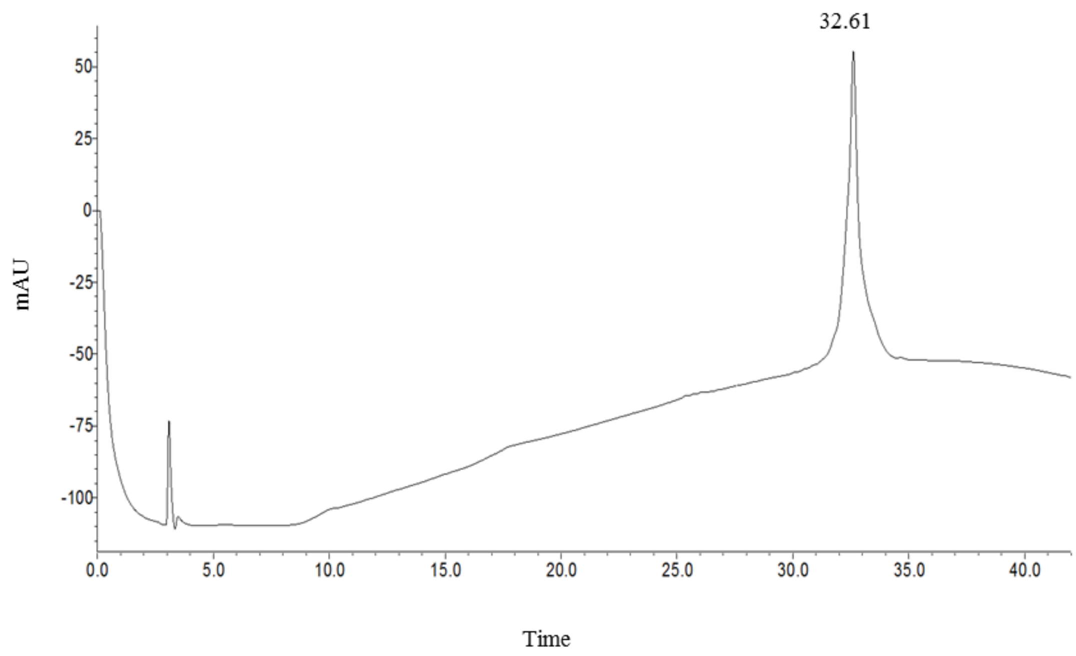

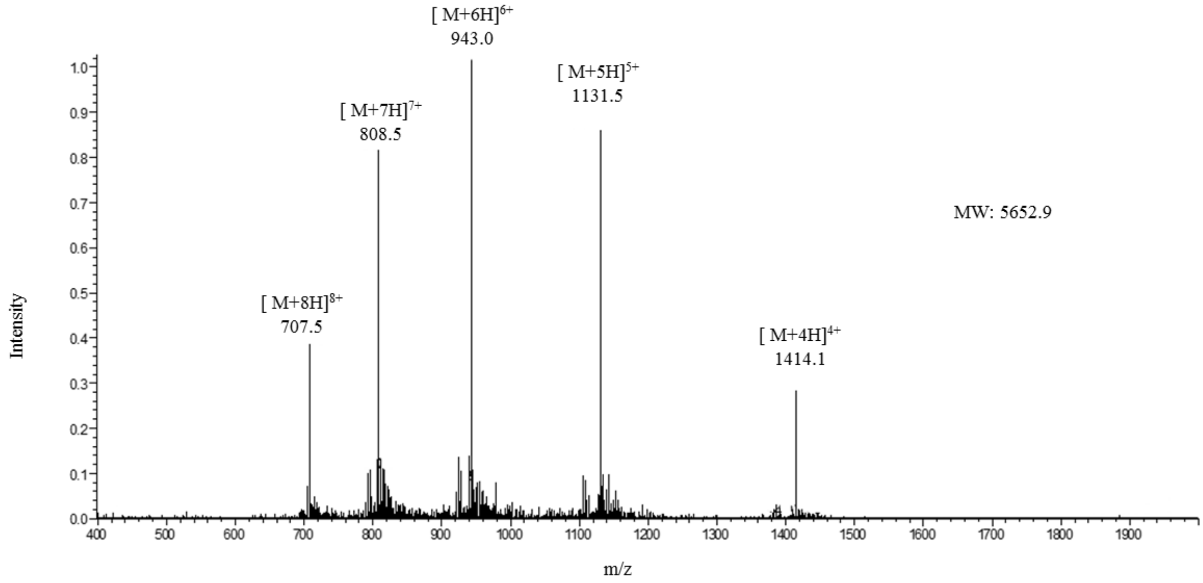

Figure S11 IILCP4. Yield: 16%. Molecular Weight: 5652.9. ESI-MS $[M+4H^{+}]^{4+}$ m/z 1414.1 (calc. 1414.2), $[M+5H^{+}]^{5+}$ m/z 1131.5 (calc. 1131.5), $[M+6H^{+}]^{6+}$ m/z 943.0 (calc. 943.1), $[M+7H^{+}]^{7+}$ m/z 808.5 (calc. 808.5), $[M+8H^{+}]^{8+}$ m/z 707.5 (calc. 707.6). t_R= 32.61 min (0-100% solvent B; C4 column), purity ≥95.

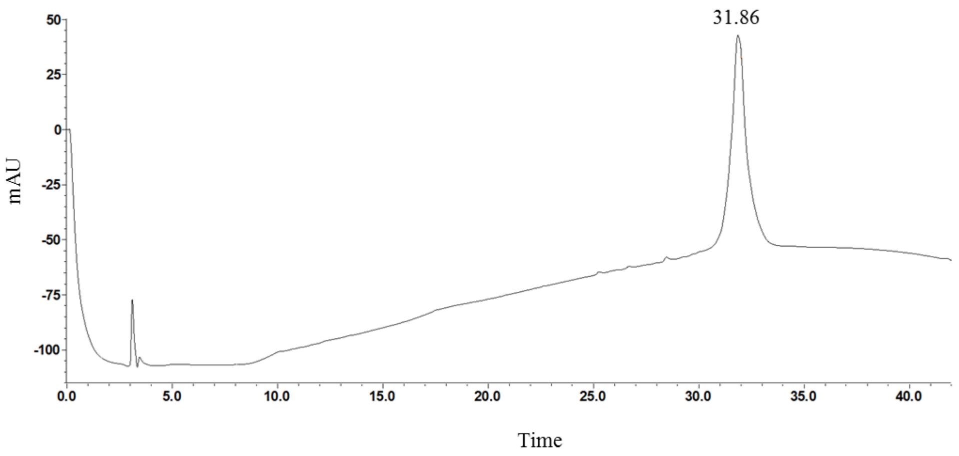

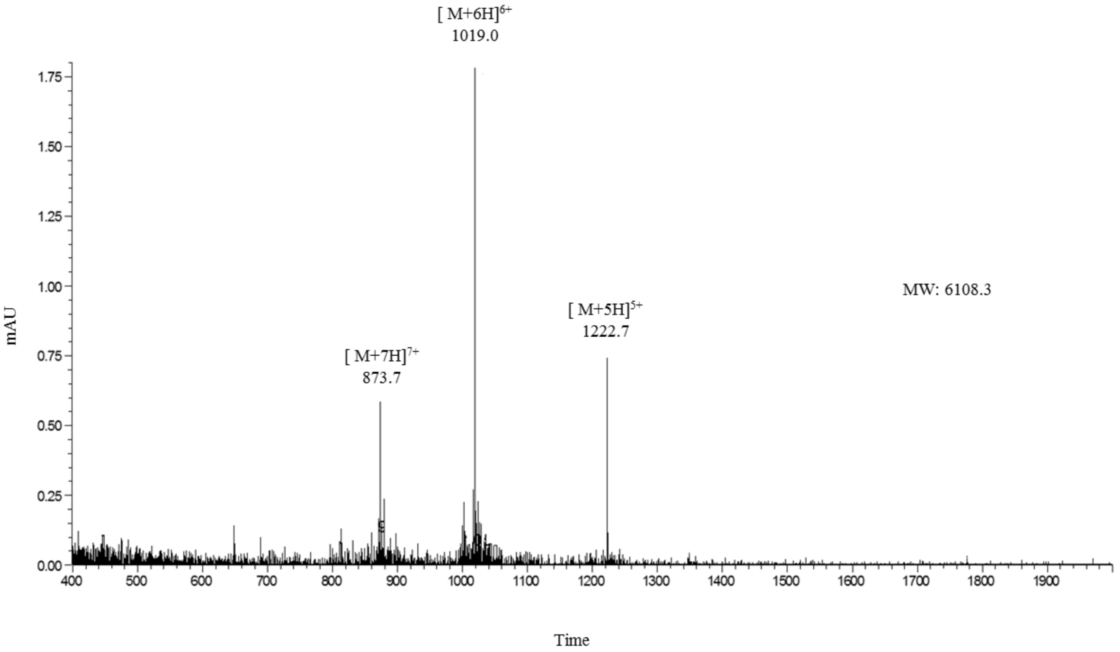

Figure S12 IILCP5. Yield: 23%. Molecular Weight: 6108.3. ESI-MS $[M+5H^+]^{5+}$ m/z 1222.7 (calc. 1222.6), $[M+6H^+]^{6+}$ m/z 1019.0 (calc. 1019.0), $[M+7H^+]^{7+}$ m/z 873.7 (calc. 873.6). t_R= 31.86 min (0-100% solvent B; C4 column), purity ≥95.

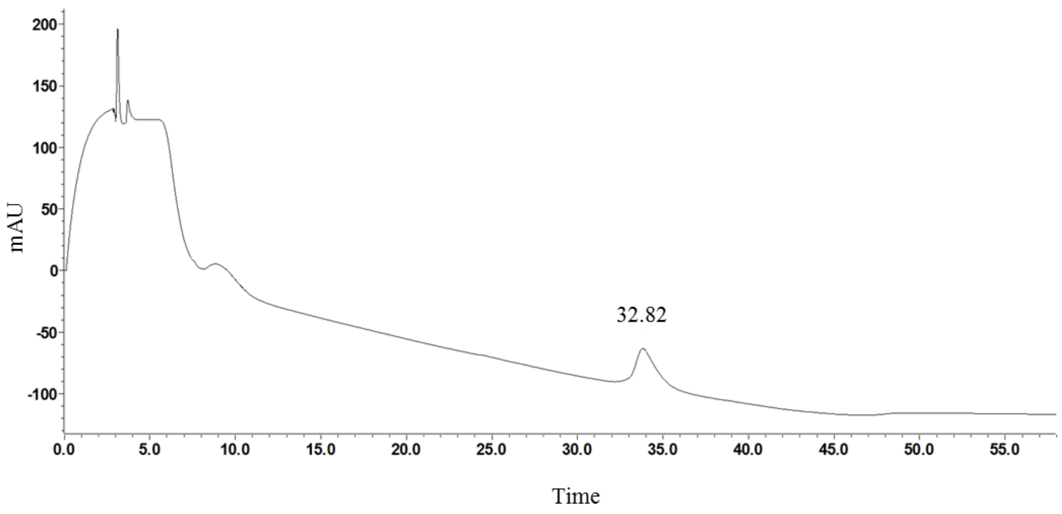

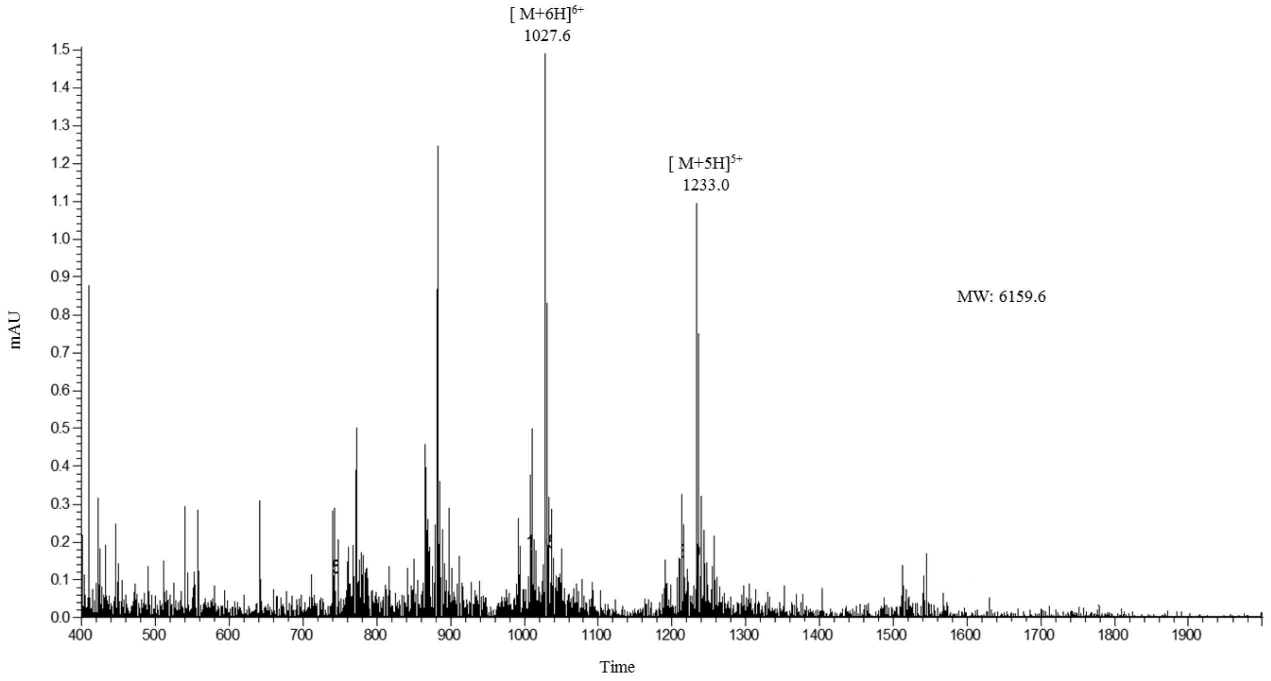

Figure S13 IILCP6. Yield: 13%. Molecular Weight: 6159.6. ESI-MS $[M+5H^{+}]^{5+}$ m/z 1233.0 (calc. 1232.9), $[M+6H^{+}]^{6+}$ m/z 1027.6 (calc. 1207.6). t_R= 32.82 min (50-100% solvent C; C4 column), purity ≥95.

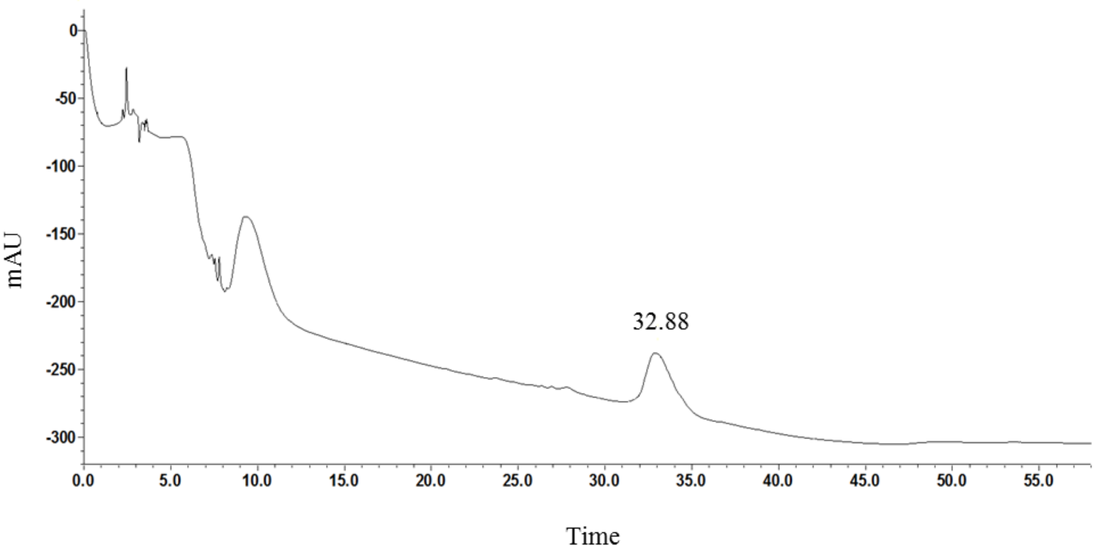

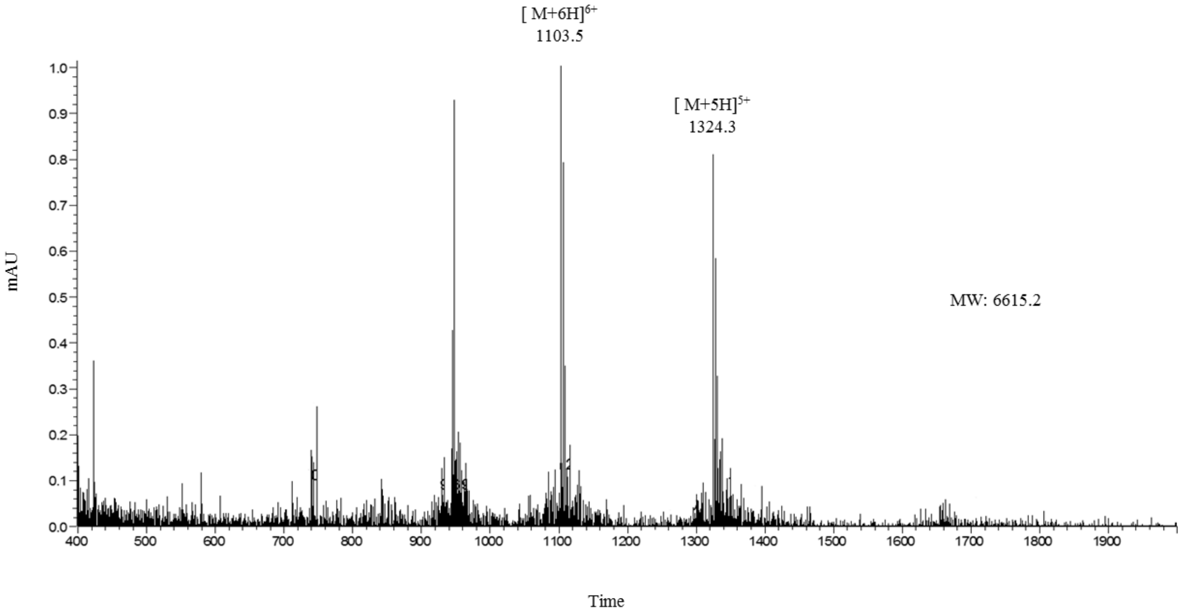

Figure S14 IILCP7. Yield: 10%. Molecular Weight: 6615.2. ESI-MS $[M+5H^+]^{5+}$ m/z 1324.3 (calc. 1324.0), $[M+6H^+]^{6+}$ m/z 1103.5 (calc. 1103.5). t_R= 32.88 min (50-100% solvent C; C4 column), purity ≥95.

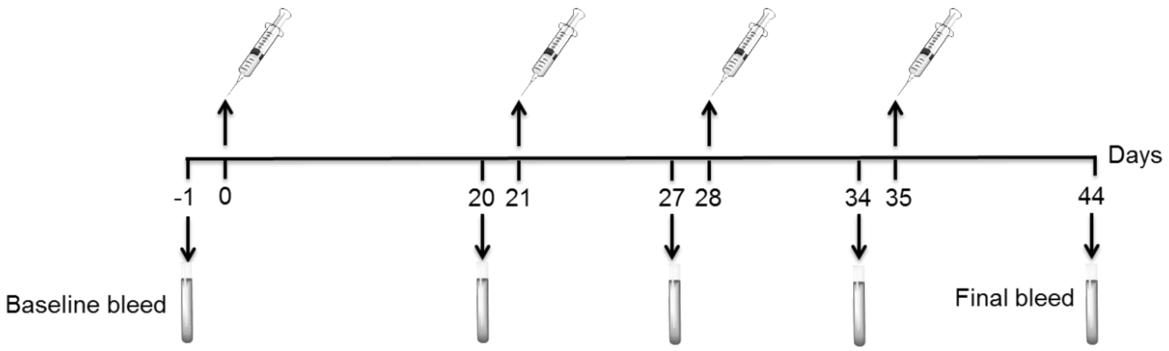

Figure S15 Experimental timeline for in vivo experiment**.** Female C57BL/6 mice were immunised by the S.C route starting at day 0. Blood samples were collected on days -1, 20, 27, 34 and 44.

A

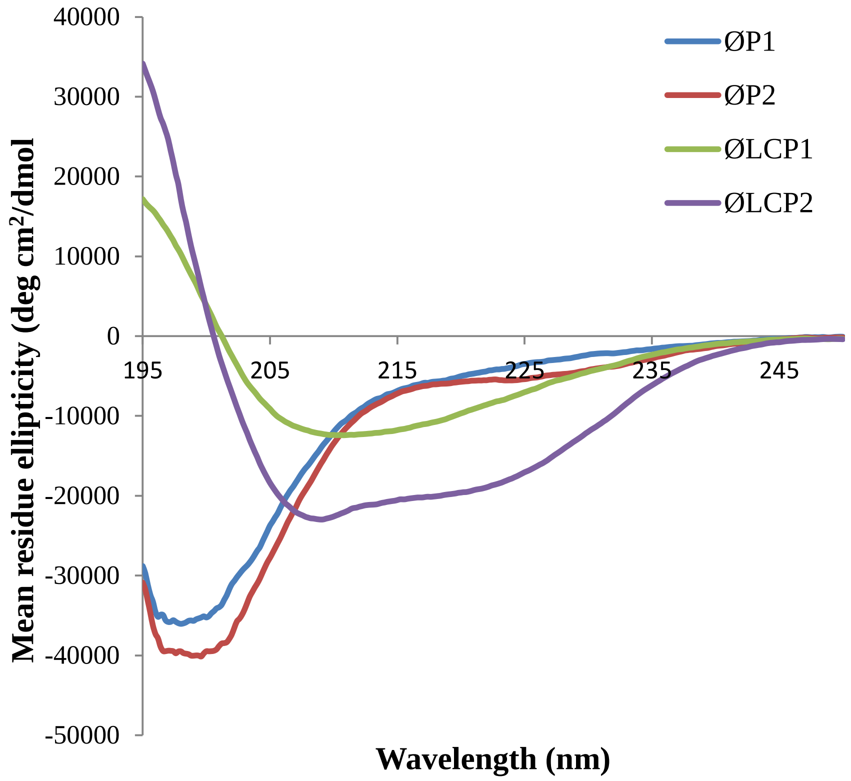

B

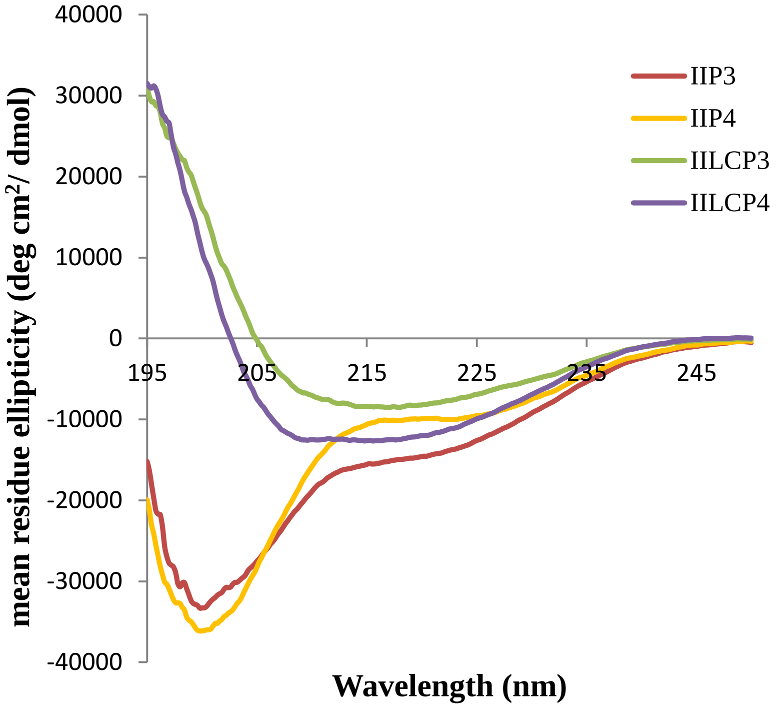

C

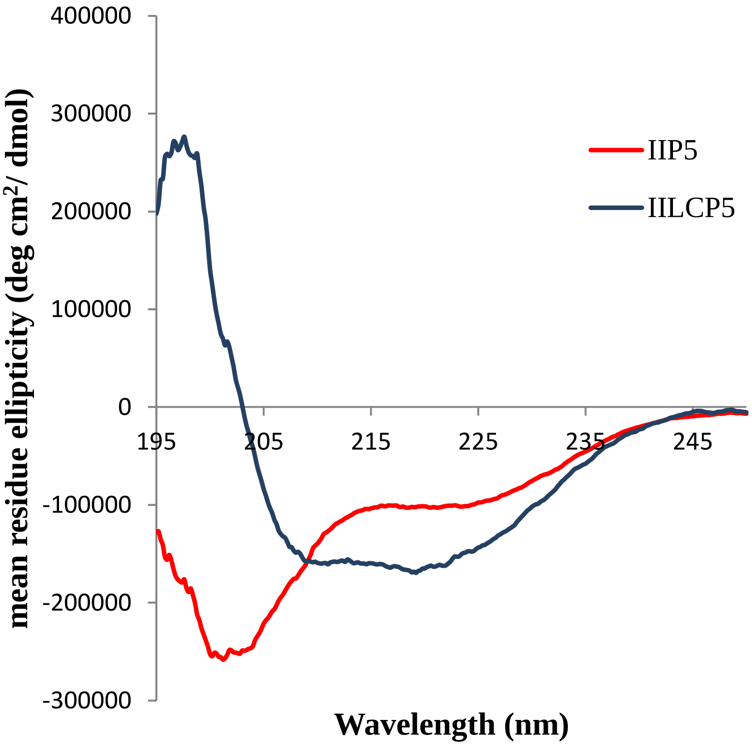

Figure S16 Circular dichroism (CD) spectra of peptides and vaccine candidates in water. CD results of constructs containing **A**) antigenic site Ø, **B**) and **C**) antigenic site II.

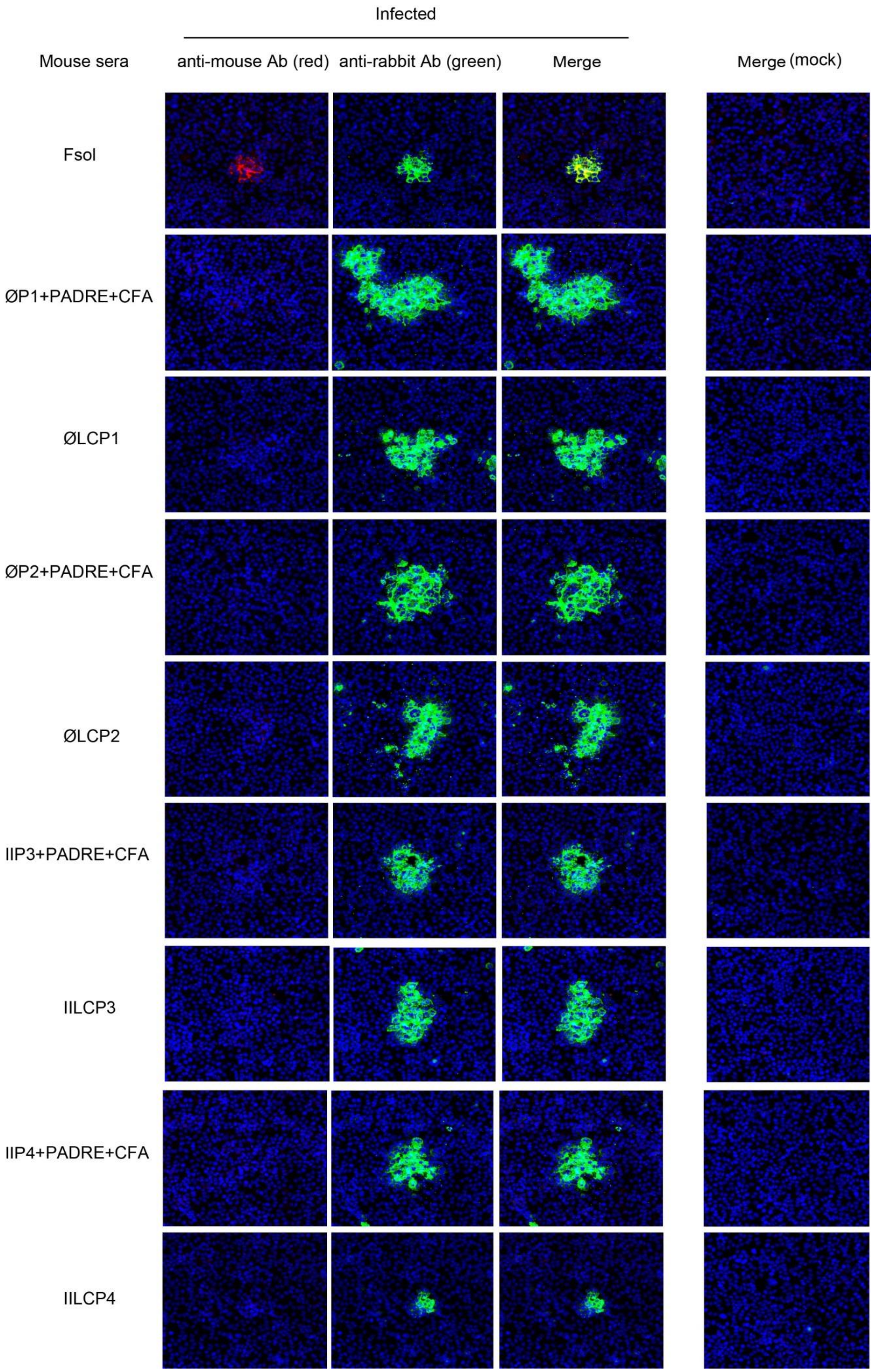

Infected
Mouse sera
anti-mouse Ab (red)
anti-rabbit Ab (green)
Merge
Merge (mock)
Fsol
ØP1+PADRE+CFA
ØLCP1
ØP2+PADRE+CFA
ØLCP2
IIP3+PADRE+CFA
IILCP3
IIP4+PADRE+CFA
IILCP4

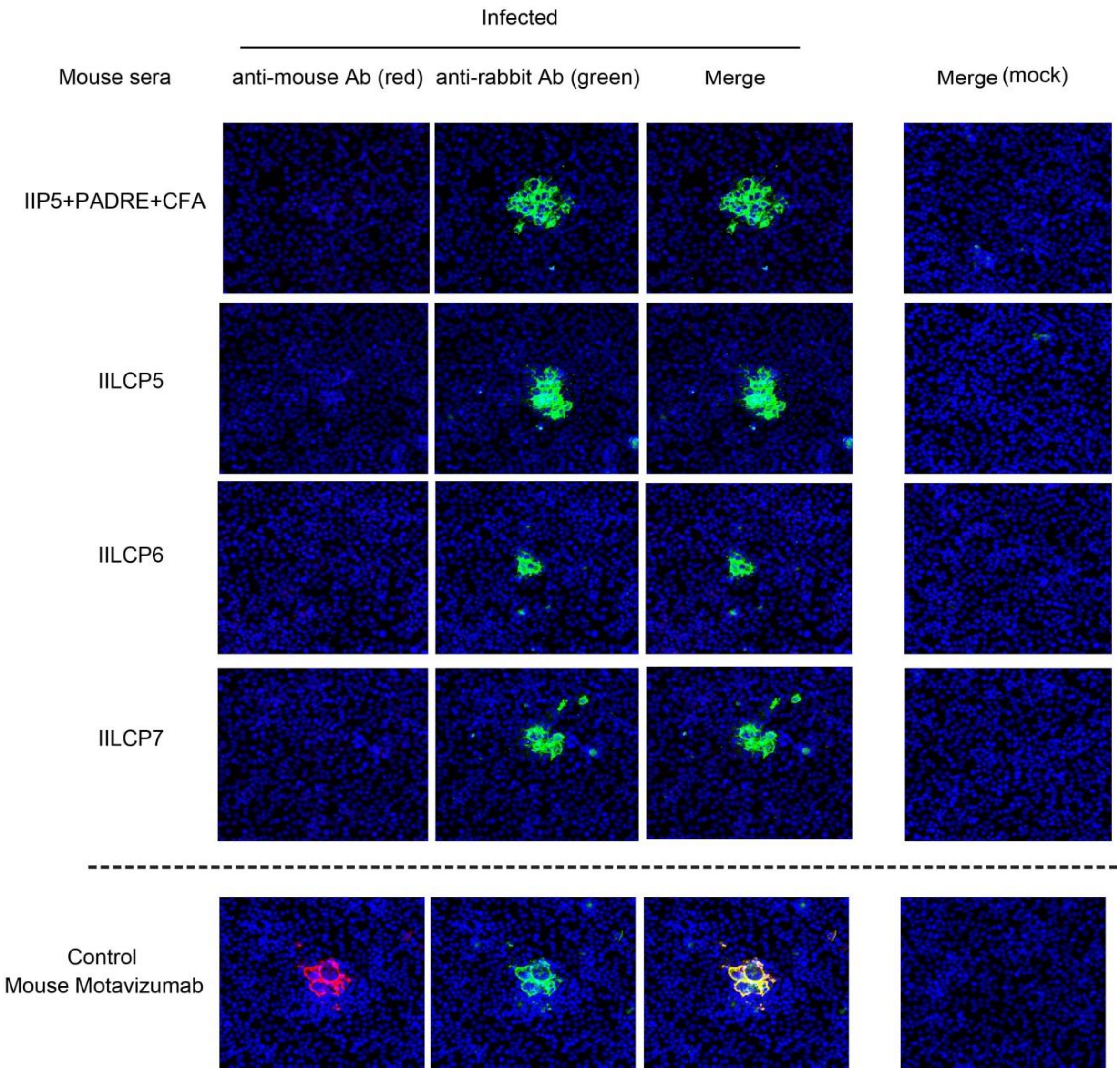

Figure S17 Binding of sera to RSV infected cells assessed using immunofluorescence. Infected and mock infected were used to assess the ability of sera from immunised mice to recognise native RSV F. To aid visualisation of the cell monolayer nuclei were stained with HOECHST dye (blue). RSV infection was confirmed with the use of a rabbit anti-RSV F polyclonal (green). The ability of vaccinated mice sera to bind to infected cell was visualised with fluorophore 555-conjugated anti-mouse antibody (red). All sera was used at a 1:20 dilution. Mouse Motavizumab was used as a positive control for reactivity (bottom panels). Analysis was performed on Zeiss LSM 710 inverted confocal microscope. Only sera from mice immunised with F_{sol} could detect RSV infection.

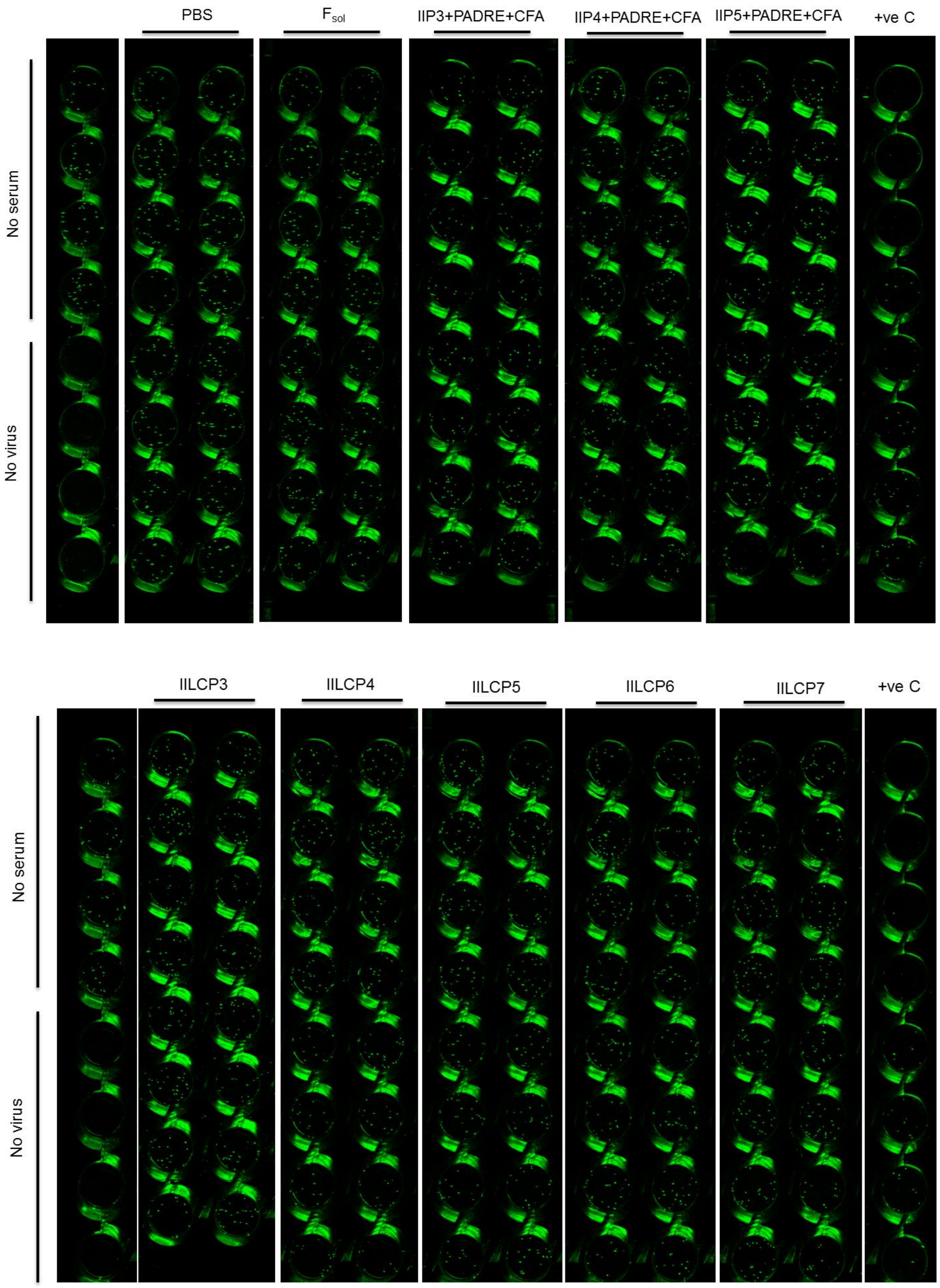
PBS
F_{sol}
IIP3+PADRE+CFA
IIP4+PADRE+CFA
IIP5+PADRE+CFA
+ve C
No serum
No virus
IILCP3
IILCP4
IILCP5
IILCP6
IILCP7
+ve C
No serum
No virus

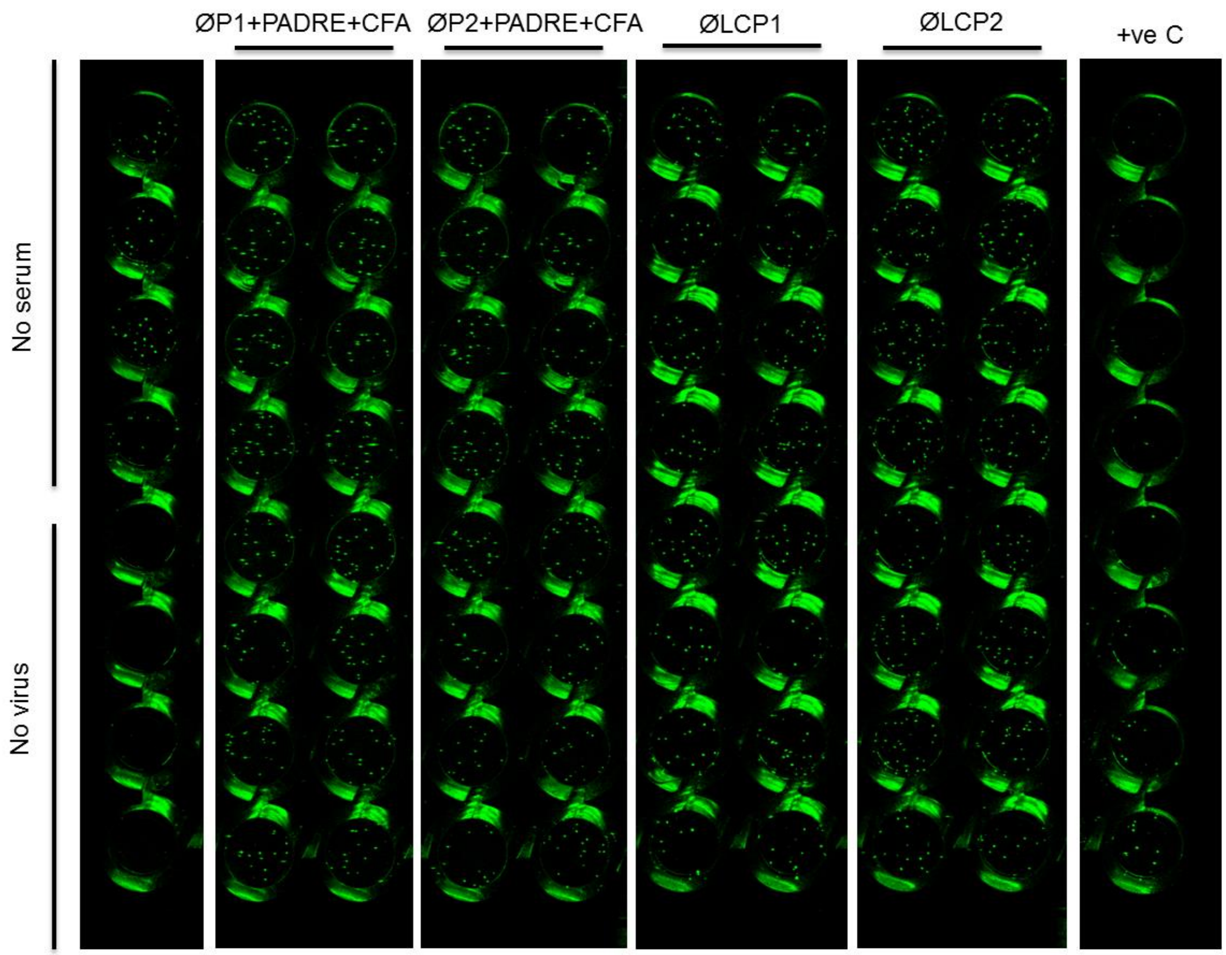

Figure S18 Plaque reduction neutralisation test. Vero cells were incubated with the serum-virus mixture. No neutralisation was observed. Positive control (+ve C) is serial dilution of Motavizumab.
